# Prion protein modulates glucose homeostasis by altering intracellular iron

**DOI:** 10.1038/s41598-018-24786-1

**Published:** 2018-04-26

**Authors:** Ajay Ashok, Neena Singh

**Affiliations:** Department of Pathology, School of Medicine, Case Western Reserve University, Cleveland, Ohio, 44106 USA

## Abstract

The prion protein (PrP^C^), a mainly neuronal protein, is known to modulate glucose homeostasis in mouse models. We explored the underlying mechanism in mouse models and the human pancreatic β-cell line 1.1B4. We report expression of PrP^C^ on mouse pancreatic β-cells, where it promoted uptake of iron through divalent-metal-transporters. Accordingly, pancreatic iron stores in PrP knockout mice (PrP^−/−^) were significantly lower than wild type (PrP^+/+^) controls. Silencing of PrP^C^ in 1.1B4 cells resulted in significant depletion of intracellular (IC) iron, and remarkably, upregulation of glucose transporter GLUT2 and insulin. Iron overloading, on the other hand, resulted in downregulation of GLUT2 and insulin in a PrP^C^-dependent manner. Similar observations were noted in the brain, liver, and neuroretina of iron overloaded PrP^+/+^ but not PrP^−/−^ mice, indicating PrP^C^-mediated modulation of insulin and glucose homeostasis through iron. Peripheral challenge with glucose and insulin revealed blunting of the response in iron-overloaded PrP^+/+^ relative to PrP^−/−^ mice, suggesting that PrP^C^-mediated modulation of IC iron influences both secretion and sensitivity of peripheral organs to insulin. These observations have implications for Alzheimer’s disease and diabetic retinopathy, known complications of type-2-diabetes associated with brain and ocular iron-dyshomeostasis.

## Introduction

Type-2-diabetes is a metabolic disorder characterized by hyperglycemia resulting from decreased secretion of insulin due to pancreatic β-cell dysfunction and resistance of peripheral organs to available insulin. The underlying pathobiology is complex, and includes a combination of host genetics and environmental factors^1^. Among the latter, a positive correlation between systemic iron and type-2-diabetes has been recognized for some time, but the underlying mechanism is not clear^2–9^. This correlation takes on an increased significance since type-2-diabetes is a known risk for Alzheimer’s disease (AD)^10,11^, a common dementia of the elderly associated with impaired neuronal glucose metabolism and brain iron dyshomeostasis^12^. Likewise, diabetic retinopathy (DR), another complication of type-2-diabetes, is fueled by iron dyshomeostasis^13^. The possibility that iron serves as the pathogenic link between type-2-diabetes, AD, and DR is intriguing, and offers untapped opportunities for a better understanding of disease pathogenesis and unconventional therapeutic options through iron chelation.

Interestingly, prion protein (PrP^C^), a mainly neuronal protein^14–16^, has been reported to influence glucose homeostasis in mouse models^17–19^ and facilitate iron uptake by functioning as a ferrireductase (FR) partner for divalent metal transporters^20^. Though apparently disconnected, it is likely that PrP^C^ modulates blood glucose by altering the expression of glucose transporter 2 (GLUT2) on pancreatic β-cells through iron, a bidirectional glucose transporter that regulates the release of insulin^21–23^. A similar function of PrP^C^ on neuronal cells might induce neurotoxicity by the combined effect of iron-mediated oxidative stress and glucose deprivation in disorders associated with brain and ocular iron dyshomeostasis such as AD, sporadic Creutzfeldt-Jakob-disease (sCJD), and DR^24^. Experimental proof of this hypothesis, however, is lacking.

Here, we explored the correlation between PrP^C^-mediated change in IC iron and expression of glucose transporters in pancreatic β-cells, hepatocytes, neuronal cells, and the retina in mouse and cell models expressing variable levels of PrP^C^ in the absence or presence of excess iron. We report that PrP^C^ influences glucose homeostasis by modulating the expression of glucose transports through iron. Implications for AD and DR, common complications of long-standing type-2-diabetes associated with brain and ocular iron dyshomeostasis are discussed.

## Results

The following mouse lines were used in this study; F2 generation of wild-type C57BL/6 (labeled as C6 PrP^+/+^) crossed with PrP-knock out on 129/Ola background developed by Manson *et al*.^25^ (labeled as C6 PrP^−/−^), and FVB/NJ PrP^−/−^ developed by Fischer *et al*. (labeled as PrP^−/−^)^26^ that were used to develop FVB/NJ Tg40 mice that express 2x human PrP (labeled as Tg40 PrP).

### PrP^C^ is expressed mainly on β-cells of mouse pancreas

**Figure 1:**
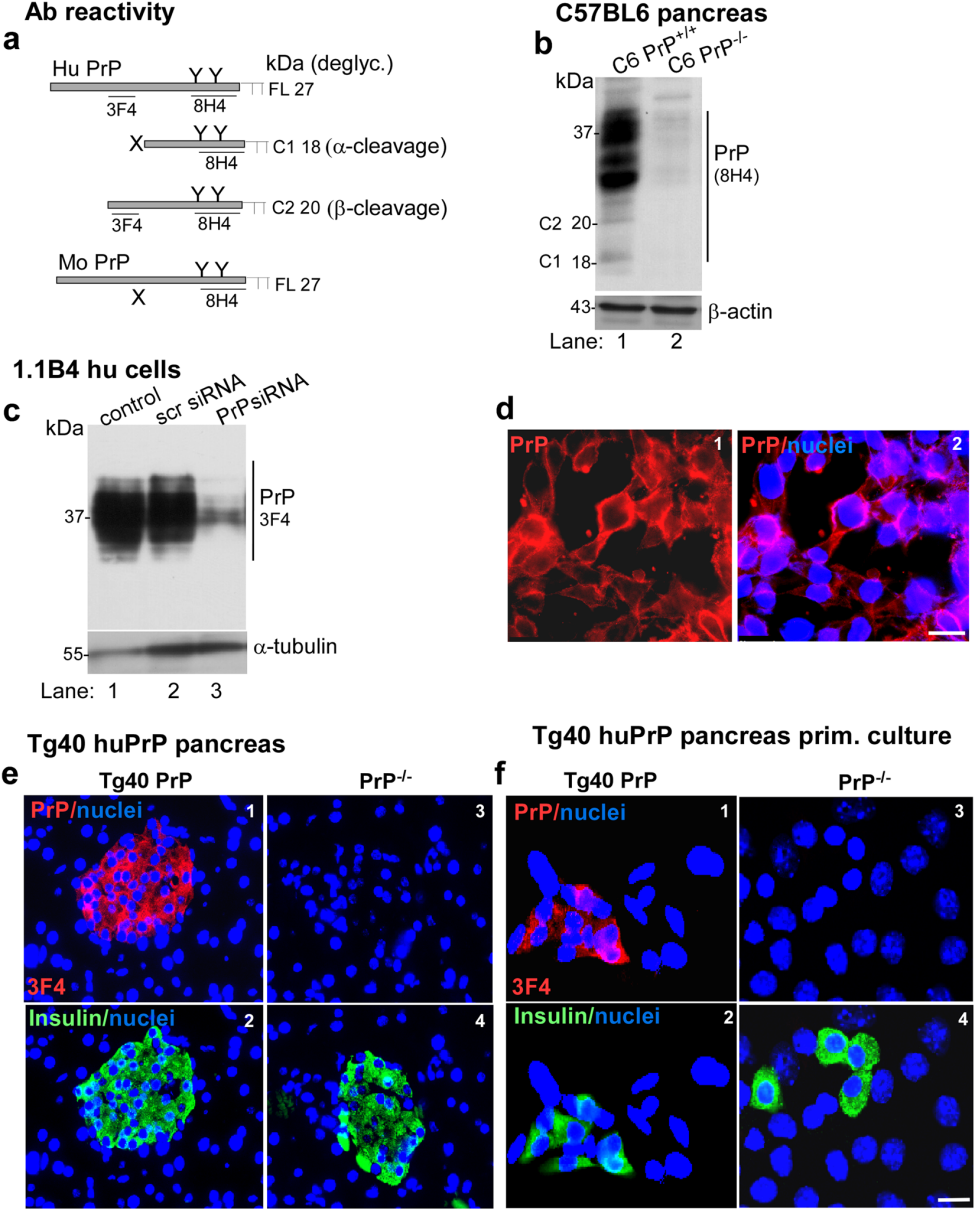
PrP is expressed on insulin positive β-cells in mouse pancreas, primary cells from mouse pancreas, and human insulin producing β-cell line 1.1B4: (**a**) Schematic representation of full length (FL), α-cleaved (C1), and β-cleaved (C2) forms of PrP and antibody reactivity. (**b**) Probing of pancreatic lysates for PrP shows the expected glycoforms in C6 PrP^+/+^ samples and no reactivity in C6 PrP^−/−^ samples (lanes 1 & 2). β-actin provides a loading control. (**c**) Probing of lysates from 1.1B4 cells for PrP shows the expected glycoforms that are down-regulated in cells transfected with PrP-specific siRNA. α-tubulin provides a loading control. The full-length blots are provided in Supplementary File (Raw data). (**d**) Immunoreaction of 1.1B4 cells for PrP shows plasma membrane and endosomal reaction (panels 1 & 2). Scale bar: 20 μm. (**e**) Immunoreaction of pancreatic sections from Tg40 PrP mice with 3F4 shows a positive reaction for PrP that co-localizes with insulin staining on β-cells (panels 1 & 2). No reaction for PrP is detected in PrP^−/−^ samples that show a robust reaction for insulin (panels 3 & 4). (**f**) Primary cultures from Tg40 PrP pancreas show a positive reaction for PrP in insulin positive β-cells (panels 1 & 2). No reaction for PrP is detected in cells from PrP^−/−^ pancreas though insulin positive β-cells are prominent (panels 3 & 4). Scale bar: 20 μm.

To evaluate the expression and post-translational processing of PrP^C^ in mouse pancreas, immunohistochemistry and Western blotting was performed with two PrP-specific antibodies; 3F4 that reacts with human PrP, not mouse PrP, and 8H4 that reacts with both human and mouse PrP. Both antibodies react with unglycosylated and post-translationally glycosylated full-length (FL) PrP forms. However, their reactivity with post-translationally processed α-cleaved (C1) and β-cleaved (C2) C-terminal fragments of PrP differs^27^. 3F4 reacts with C2, not C1 where its epitope is lost, whereas 8H4 reacts with both C1 and C2. Thus, differential antibody reactivity combined with migration on SDS gels based on molecular mass provides a convenient method for distinguishing between α- (C1) and β-cleaved (C2) forms of PrP (Fig. 1a).

Probing of Western blots of pancreatic lysates from C6 PrP^+/+^ and C6 PrP^−/−^ mice with 8H4 revealed unglycosylated and glycosylated FL, and C2 and C1 forms similar to neuronal PrP^C ^^27^ in C6 PrP^+/+^, and no reactivity in C6 PrP^−/−^ samples (Fig. 1b, lanes 1 & 2). Probing of 1.1B4 cell lysates with 3F4 revealed the unglycosylated and glycosylated FL PrP^C^ as above, and almost complete loss of reactivity by silencing PrP (Fig. 1c, lanes 1–3). Immunoreaction of permeabilized 1.1B4 cells with 3F4 followed by Alexa Fluor-conjugated secondary antibody revealed prominent reaction on the plasma membrane and endocytic vesicles as in neuronal cells (Fig. 1d, panels 1 & 2)^28^.

Immunoreaction of fixed sections of pancreas from Tg40 PrP and matching PrP^−/−^ mice with 3F4 and insulin-specific antibody showed a strong reaction for PrP^C^ on insulin positive β-cells in Tg40 PrP samples, and a positive reaction for insulin, but not 3F4 in PrP^−/−^ samples (Fig. 1e, panels 1–4). A similar evaluation of cultured primary cells from freshly harvested pancreas showed a positive reaction for PrP^C^ in insulin positive cells from Tg40 PrP samples, a reaction for insulin in PrP^−/−^ samples that did not react with 3F4 as expected (Fig. 1f, panels 1–4).

### Most of the PrP^C^ in pancreatic β-cells is cleaved at the β-site

Under physiological conditions, PrP^C^ expressed on neuronal cells recycles from the plasma membrane and undergoes α-cleavage in an endocytic compartment, resulting in the generation of C1^27^. Cleavage at the β-site is typical of disease-associated PrP-scrapie (PrP^Sc^)^14,29–31^, and is triggered by exposure to reactive oxygen species^32^.

**Figure 2:**
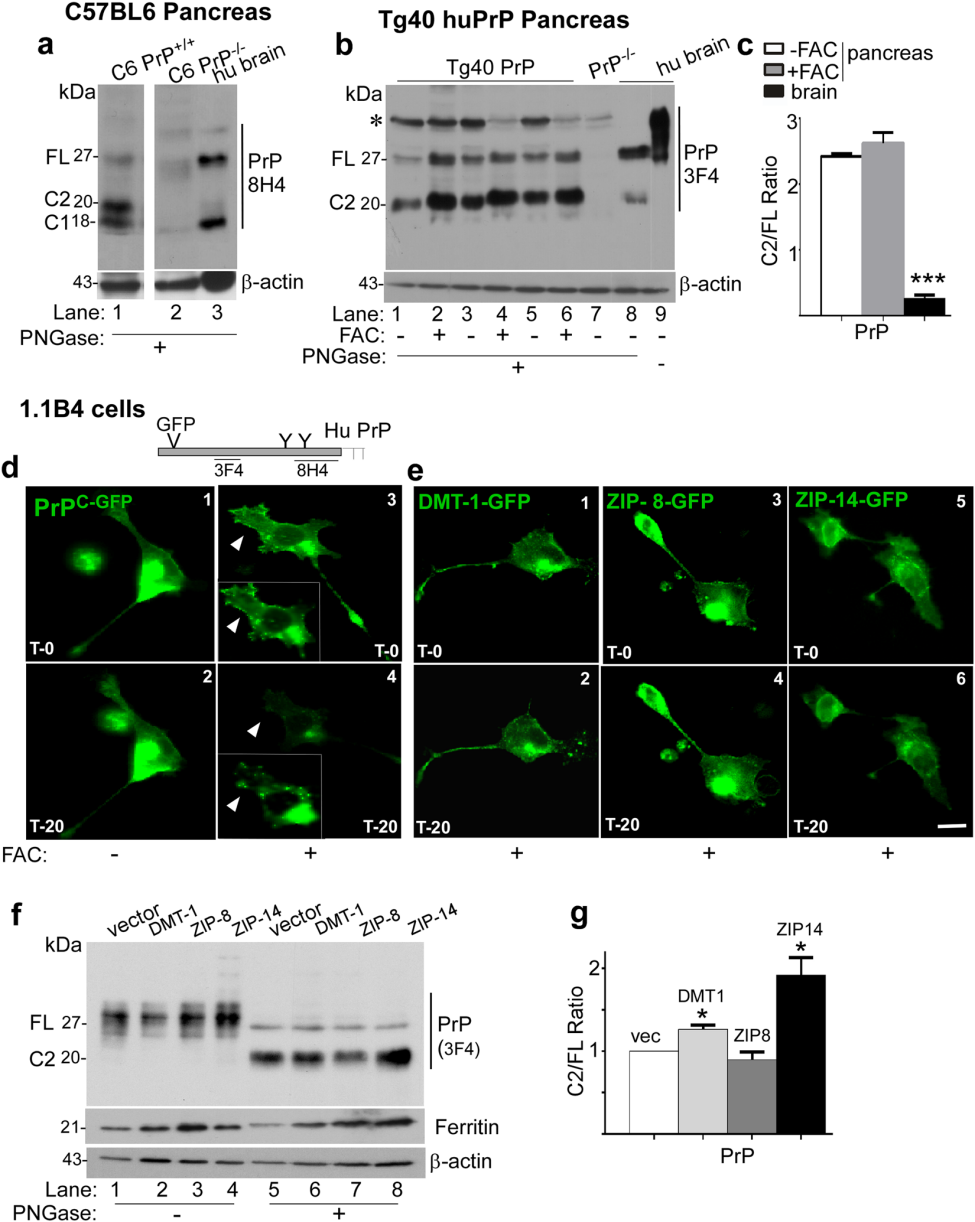
Iron uptake is coupled with β-cleavage of PrP^C^: **(a)** Probing of deglycosylated pancreatic lysates from C6 PrP^+/+^ mice with 8H4 shows β-cleavage of majority of PrP. No reactivity is detected in C6 PrP^−/−^ samples as expected. Most of the PrP from human brain homogenate is cleaved at the α-site (lanes 3). (All samples were fractionated and processed on the same gel. Complete gel is included in supplementary file-raw data). *Represents a non-specific band. **(b)** Probing of deglycosylated pancreatic lysates from Tg40 PrP mice with 3F4 shows β-cleavage of majority of PrP (lanes 1–6). Systemic iron overload increases FL and the β-cleaved form of PrP (lanes 2, 4 & 6 vs. 1, 3 & 5). Most of the PrP in human brain homogenate is FL (lanes 8 & 9). Pancreatic lysates from PrP^−/−^ mice show no reactivity for 3F4 as expected. **(c)** The ratio of C2 vs. full-length PrP is significantly higher in pancreatic lysates relative to the brain. Values are mean + SEM of the indicated n. ***p < 0.001. **(d)** Exposure of PrP-GFP expressing 1.1B4 cells to ferric ammonium citrate (FAC) results in the loss of GFP tag from the plasma membrane within 20 minutes (panels 1–4). **(e)** Exposure of 1.1B4 cells expressing GFP-tagged DMT-1, ZIP8, or ZIP14 to FAC, on the other hand, shows no change in localization or intensity of GFP fluorescence up to 20 minutes (panels 1–6). Scale bar 20 μm. **(f)** Probing of deglycosylated lysates from 1.1B4 cells expressing vector, DMT-1, ZIP8, and ZIP14 with 3F4 reveals significant increase in the ratio of C2/FL in cells expressing DMT-1 and ZIP14 (lane 5 vs. 6 & 8; **g**). (The expression of GFP-tagged transfected proteins was equivalent (at 15–17%) as determined by manual counting of 20 random fields of each at 5×)- **(**see Supplementary Fig. S3**)**. **(g)** The ratio of C2 vs. FL after normalization with transfection efficiency and β-actin shows a significant increase in cells expressing DMT-1 and ZIP14. Values are mean + SEM of the indicated n. *p < 0.05. The full-length blots are provided in Supplementary File (Raw data).

To evaluate whether post-translational processing of PrP^C^ on pancreatic β-cells differs from neurons^27,33^, pancreatic lysates from C6 mice and human brain (as a representative tissue for neurons) were deglycosylated and probed with 8H4. Surprisingly, pancreatic lysates from C6 PrP^+/+^ samples revealed barely detectable FL PrP^C^, most of which was detected as C2 and C1 fragments. Human brain sample, on the other hand, showed equivalent amounts of FL and C1, and C6 PrP^−/−^ samples did not show any reactivity as expected (Fig. 2a, lanes 1–3).

Similar results were obtained from deglycosylated pancreatic lysates from Tg40 PrP mice probed with 3F4. Majority of PrP^C^ in the pancreas exhibited the mobility of C2. Systemic iron overload induced by injecting 36 µg/22 g mouse weight ferric ammonium citrate (FAC) upregulated FL PrP^C^ to some extent, but the major difference was in the intensity of C2. (Fig. 2b, lanes 1–6; Fig. 2c). Pancreatic lysates from PrP^−/−^ mice showed no reactivity with 3F4, while brain lysates revealed mainly FL PrP^C^ and minimal reactivity for C2 (Fig. 2b, lanes 7–9; Fig. 2c). The small increase in C2 in iron over-loaded pancreas (Fig. 2c) indicated iron-mediated cleavage of PrP *in vivo*, a phenomenon that was investigated further in the 1.1 B4 cells *in vitro*. Similar results were obtained from probing the membrane with 8H4 (see Supplementary Fig. S2).

Accordingly, 1.1B4 cells expressing green-fluorescent protein (GFP) tagged PrP^C^ (PrP^C-GFP^) were exposed to FAC, and live cells were imaged every 5 min for up to 20 min. Since the GFP tag is inserted between N-terminal residues 37 and 38 of PrP^20,34^, the premise was that the tag would be lost following α- or β-cleavage of PrP^C^. Representative images from time zero (T-0) and 20 min (T-20) time point are shown (Fig. 2d, panels 1–4). In control cells exposed to vehicle, the signal from PrP^C-GFP^ was prominent at the plasma membrane, peri-nuclear Golgi region, and endocytic vesicles at T-0 and T-20 (Fig. 2d, panels 1 & 2). In FAC exposed cells, the distribution of PrP^C-GFP^ at T-0 was similar to controls (Fig. 2d, panels 1 & 3). However, at T-20 most of the GFP signal was lost from the plasma membrane, though peri-nuclear and endosomal signal remained (Fig. 2d, panel 4). (The intensity of green fluorescence in the inset was increased 5-fold in panels 3 and 4). Thus, the GFP carrying N-terminus of PrP^C^ is lost within 20 min of exposure to FAC. Based on Western blot results in Fig. 2b and published reports^32,35^, it is likely that FAC induces β-cleavage of PrP^C^.

To evaluate whether β-cleavage of PrP^C^ is coupled with iron transport through divalent metal transporter-1 (DMT-1) or members of the Zrt Irt-like protein (ZIP) family, in particular ZIP14^20,36,37^, 1.1B4 cells transfected with GFP-tagged DMT-1, ZIP8, and ZIP14 were exposed to FAC and imaged at T-0 and T-20. Unlike PrP^C-GFP^ (Fig. 2d), there was no difference in the intensity or localization of DMT-1-GFP, ZIP8-GFP, or ZIP14-GFP following exposure to exogenous FAC (Fig. 2e, panels 1–6). However, overexpression of these transporters in 1.1B4 cells resulted in increased β-cleavage of PrP^C^ in cells transfected with DMT-1 and ZIP14 (Fig. 2f, lanes 6 & 8; Fig. 2g). Since ZIP14 is known to mediate uptake of non-transferrin bound iron (NTBI) by pancreatic β-cells^38^ (see Supplementary Fig. S1), these results suggest that PrP^C^ functions as a FR partner for ZIP14, and is cleaved at the β-site during this process.

### PrP^C^ mediates uptake of iron by pancreatic β-cells *in vitro* and *in vivo*

**Figure 3:**
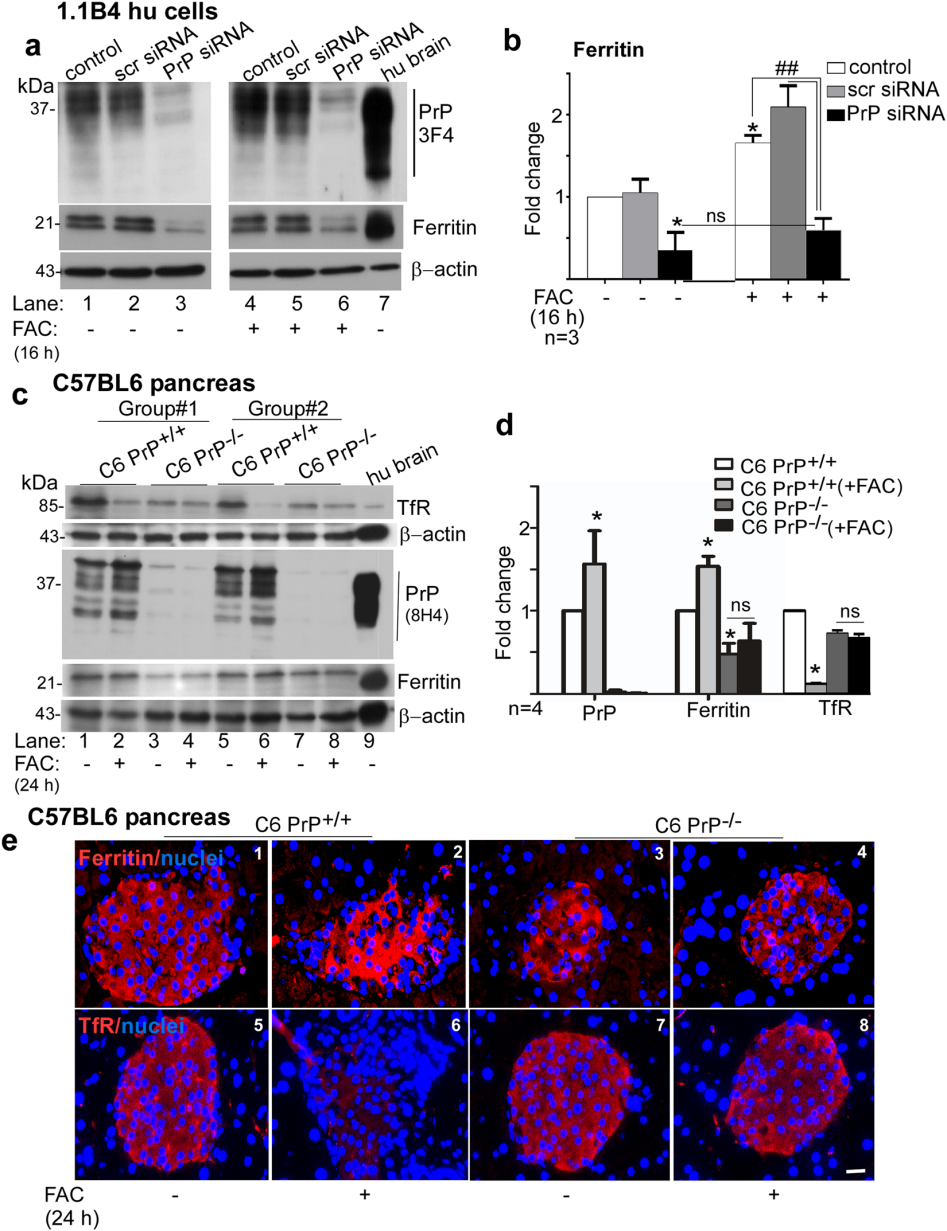
PrP mediates iron uptake by pancreatic β-cells: **(a)** Non-transfected and 1.1B4 cells transfected with scrambled or PrP-specific siRNA were cultured in the absence (−FAC) or presence of ferric ammonium citrate (+FAC) for 16 h, and lysates were processed for Western blotting. Probing with 3F4 shows PrP glycoforms, and down-regulation by PrP-specific siRNA. Probing for ferritin shows significant down-regulation in cells where PrP had been silenced. Exposure to exogenous iron upregulates ferritin in untreated controls and cells transfected with scrambled siRNA, but has minimal effect on cells where PrP is silenced. **(b)** Quantification of ferritin expression by densitometry after normalization with β-actin. Values are mean ± SEM of the indicated n. *Represents change in ferritin relative to untreated, non-transfected control. ^##^Represents change in ferritin relative to FAC exposed non-transfected control. *p < 0.05, ^##^*p* < 0.01. **(c)** Western blotting shows upregulation of PrP in iron-overloaded C6 PrP^+/+^, no signal in C6 PrP^−/−^ samples, and the expected glycoforms in human brain sample (lane 9). Ferritin is significantly higher in iron-overloaded relative to untreated C6 PrP^+/+^ samples and matched C6 PrP^−/−^ samples. There is no change in ferritin iron overloaded C6 PrP^−/−^ samples relative to untreated controls. Probing for TfR shows significant reduction in iron-overloaded relative to untreated C6 PrP^+/+^ samples and matched C6 PrP^−/−^ samples. There is minimal change in TfR expression in iron-overloaded C6 PrP^−/−^ samples relative to untreated controls. **(d)** Density of protein bands after normalization with β-actin. Values are mean ± SEM of the indicated n. *Represents change in expression relative to untreated C6 PrP^+/+^ samples. *p < 0.05. Full-length blots are provided in Supplementary File (Raw data). **(e)** Immunoreaction of fixed pancreatic sections from the above mice for ferritin shows a positive reaction in mainly β-cell rich endocrine islets (panels 1–4). Iron over-loading increases ferritin reactivity in C6 PrP^+/+^ sections, but shows minimal change in C6 PrP^−/−^ samples (panels 3 & 4). Reactivity for TfR is also localized to the endocrine islets (panels 5–8). Iron-overloading down-regulates TfR expression in C6 PrP^+/+^ samples (panel 6 vs. 5), but has minimal effect on C6 PrP^−/−^ samples (panels 7 & 8). Scale bar 20 μm.

To confirm the facilitative role of PrP^C^ in iron uptake by the pancreas^39–41^, 1.1B4 cells were transfected with siRNA to silence PrP^C^, and exposed to FAC. Following an incubation of 16 h, control and experimental cell lysates were processed for Western blotting and probed for ferritin. Silencing of PrP^C^ resulted in significant downregulation of ferritin relative to non-transfected and scrambled siRNA transfected controls (Fig. 3a, lanes 1–3; Fig. 3b). Exposure to FAC caused significant upregulation of ferritin in controls as expected, but had minimal effect in the absence of PrP^C^ (Fig. 3a, lanes 4–6); Fig. 3b).

The above observations were substantiated in C6 PrP^+/+^ and C6 PrP^−/−^ mice injected with iron to create systemic iron overload (Fig. 3c–e). Evaluation of pancreatic lysates by Western blotting revealed upregulation of PrP^C^ in iron-overloaded C6 PrP^+/+^ mice as in Fig. 2b above (Fig. 3c, lanes 1, 2, 5, 6; Fig. 3d). Probing for ferritin revealed significantly less ferritin in C6 PrP^−/−^ relative to C6 PrP^+/+^ controls. Overloading with iron caused significant upregulation of ferritin in C6 PrP^+/+^, but not in C6 PrP^−/−^ samples (Fig. 3c, lanes 1–8; Fig. 3d). Expression of transferrin receptor (TfR) was reduced in iron overloaded C6 PrP^+/+^ mice, but showed minimal change in similarly treated C6 PrP^−/−^ mice (Fig. 3c, lanes 1–8; Fig. 3d). Similar results were observed in the Tg40 PrP and PrP^−/−^ mice (see Supplementary Fig. S4).

Immunohistochemistry of fixed sections of pancreas from the above mice revealed reactivity for ferritin in the islets of both C6 PrP^+/+^ and C6 PrP^−/−^ samples (Fig. 3e, panels 1 & 3), and a significant increase in iron overloaded C6 PrP^+/+^ relative to C6 PrP^−/−^ samples (Fig. 3e, panels 2 & 4). Reaction for (TfR) showed significant downregulation in iron overloaded C6 PrP^+/+^, but minimal change in C6 PrP^−/−^ samples (Fig. 3e, panels 5–8).

Together, the above results leave little doubt that PrP^C^ mediates iron uptake in pancreatic β-cells. Since increased systemic iron is associated with the risk of type-2-diabetes, further studies were directed at whether PrP-mediated change in pancreatic β-cell iron influences insulin production and/or secretion and blood glucose levels.

### PrP^C^-mediated increase in intracellular iron downregulates glucose transporters in the pancreas, liver, and retina

**Figure 4:**
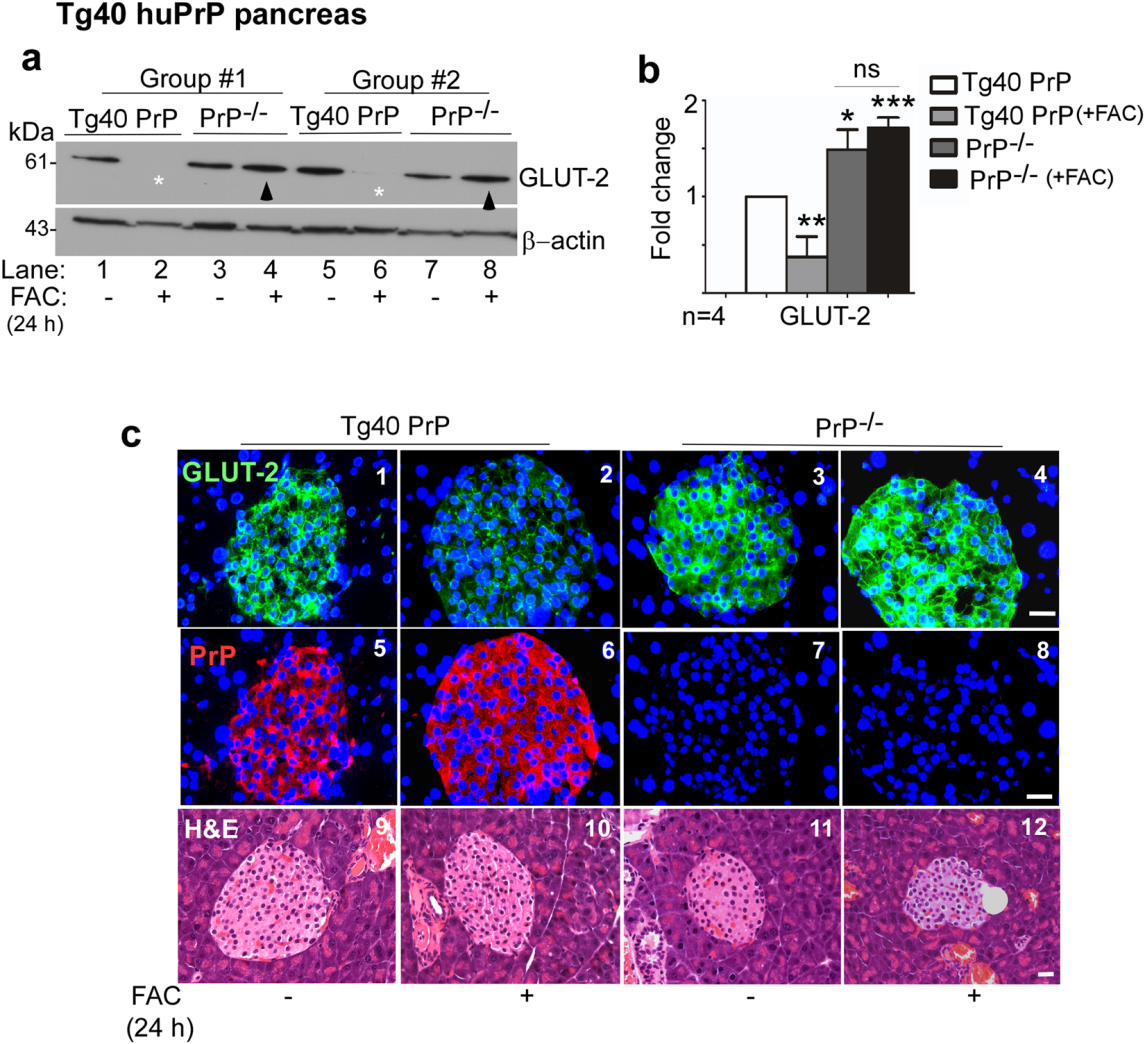
PrP-mediated increase in pancreatic β-cell iron downregulates GLUT2: **(a)** Pancreatic lysates from control and iron overloaded mice were subjected to Western blotting and probed for GLUT2. Samples from control PrP^−/−^ samples show significant upregulation of GLUT2 relative to Tg40 PrP samples (lanes 3 & 7 vs. 1 & 5; B). Iron overloading downregulates GLUT2 in Tg40 PrP samples (*star), but has minimal effect on similarly treated PrP^−/−^ samples (arrowhead) (lanes 2 & 6 vs. 4 & 8; B). **(b)** Densitometric analysis of GLUT2 expression after normalization with β-actin. Values are mean ± SEM of the indicated n. *p < 0.05, **p < 0.01, ***p < 0.001, ns, not significant. The full-length blots are provided in Supplementary File (Raw data). **(c)** Immunoreaction of pancreatic sections from the above mice mirrors the results in panel a. Immunoreaction for GLUT2 is higher in PrP^−/−^ relative to Tg40 PrP samples (panels 1 & 3). Iron overloading reduces reactivity for GLUT2 in Tg40 PrP, but not in PrP^−/−^ samples (panels 1 vs. 2 & 3 vs. 4). Reaction for PrP is limited to pancreatic islets as for GLUT2, and iron overloading upregulates PrP (panels 5 & 6). PrP^−/−^ samples show no reactivity for PrP as expected (panels 7 & 8). H&E sections show no obvious toxicity due to iron overloading (panels 9–12). Scale bar 20 μm.

Pancreatic β-cells sense blood glucose levels through GLUT2, a bidirectional glucose transporter, and release insulin to maintain glucose concentrations within a defined range^22,23^. To evaluate whether PrP^C^-mediated increase in β-cell iron alters the expression of GLUT2 (Fig. 4) and insulin (Fig. 6), pancreas from iron over-loaded Tg40 PrP and corresponding PrP^−/−^ mice were subjected to Western blotting and immunohistochemistry (Fig. 4a–c).

Probing of pancreatic lysates for GLUT2 revealed significantly higher expression in PrP^−/−^ relative to Tg40 PrP samples. Overloading with iron downregulated GLUT2 in Tg40 PrP, but had no influence on PrP^−/−^ mice (Fig. 4a, lanes 1–8, Fig. 4b).

Immunohistochemistry of pancreas mirrored the Western blot results. Iron overloading decreased GLUT2 reactivity in Tg40 PrP, but had minimal effect on PrP^−/−^ samples (Fig. 4c, panel 1–4). Expression of PrP^C^ was upregulated by excess iron as in Fig. 2 above, and PrP^−/−^ samples showed no reactivity as expected (Fig. 4c, panels 5–8). Staining with H & E showed no damage or change in the architecture of pancreatic tissue due to iron overloading (Fig. 4c, panels 9–12).

**Figure 5:**
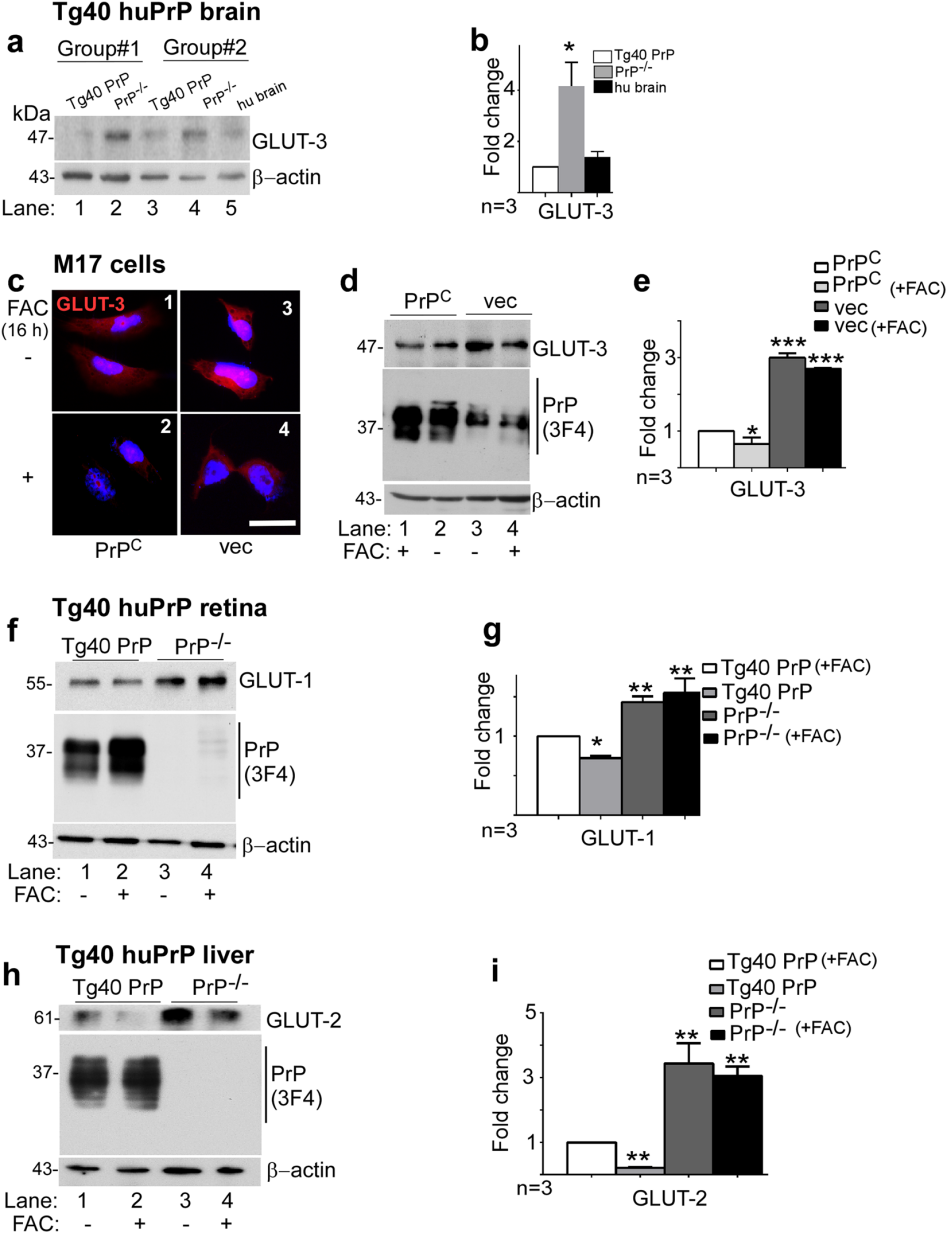
PrP-mediated increase in IC iron downregulates glucose transporters in the brain, neuroretina, and the liver: **(a)** Probing of Western blots of brain lysates from Tg40 PrP and PrP^−/−^ mice for GLUT3 shows a significant increase in PrP^−/−^ relative to Tg40 PrP samples (lanes 2 & 4 vs. 1 & 3). **(b)** Density of protein bands after normalization with β-actin. Values are mean ± SEM of the indicated n. *p < 0.05. **(c)** Immunoreaction of M17 cells for GLUT3 shows increased reactivity in vector-transfected relative to PrP^C^ over-expressing cells (panels 1 & 3). Exposure to iron downregulates GLUT3 significantly more in PrP^C^ relative to vector controls (panels 2 & 4). Scale bar 25 μm. **(d)** Western blotting of lysates from control and iron-exposed PrP^C^ and vector expressing cells mirrors the results in panel c. **(e)** Density of protein bands after normalization with β-actin. Values are mean ± SEM of the indicated n. *p < 0.05, ***p < 0.001, ns, not significant. **(f)** A similar evaluation of retinal lysates shows upregulation of GLUT1 in PrP^−/−^ relative to Tg40 PrP samples (lanes 1 & 3). Iron overloading downregulates GLUT1 in Tg40 PrP samples relative to untreated controls, but has minimal effect on similarly treated PrP^−/−^ samples (lanes 2 & 4). **(g)** Density of protein bands after normalization with β-actin. Values are mean ± SEM of the indicated n. *p < 0.05, **p < 0.01, ns, not significant. **(h)** Probing of Western blots of liver lysates for GLUT2 mirrors the results in pancreatic, brain, and neuroretinal lysates (lanes 1–4). **(i)** Density of protein bands after normalization with β-actin. Values are mean ± SEM of the indicated n. *p < 0.05, **p < 0.01, ns, not significant. The full-length blots are provided in Supplementary File (Raw data).

To evaluate whether PrP^C^-mediated increase in IC iron alters glucose transporters on neuronal cells, in particular glucose transporter 3 (GLUT3)^42^, brain lysates from Tg40 PrP and PrP^−/−^ mice were subjected to Western blotting and probed for GLUT3. As noted in pancreatic β-cells, expression of GLUT3 was upregulated in PrP^−/−^ relative to Tg40 PrP samples (Fig. 5a, lanes 1–4; Fig. 5b).

Further confirmation of this phenomenon was obtained in M17 cells, a neuroblastoma cell line transfected to over-expressing PrP^C^ and the respective vector control. Immunoreaction of fixed, permeabilized cells for GLUT3 revealed significantly less reactivity in PrP^C^-expressing cells relative to vector controls (Fig. 5c, panels 1 & 2). Exposure to exogenous iron decreased GLUT3 reactivity in both cell lines, but significantly more in PrP^C^-expressing cells relative to vector controls (Fig. 5c, panels 3 & 4). These results were confirmed by Western blotting of similarly treated cell lysates. Probing for GLUT3 revealed significantly lower expression in PrP^C^-expressing cells relative to vector controls (Fig. 5d, lanes 2 & 3; Fig. 5e). Exposure to exogenous iron downregulated GLUT3 in both cell lines, but significantly more in PrP^C^- expressing cells relative to vector controls (Fig. 5d, lanes 1 & 4; Fig. 5e).

Evaluation of samples from the liver and neuroretina of Tg40 PrP and PrP^−/−^ mice mimicked the results obtained in the pancreas, the brain, and neuronal cells. Western blotting showed downregulation of glucose transporter 1 (GLUT1) in the neuroretina and GLUT2 in the liver of Tg40 PrP relative to PrP^−/−^ samples (Fig. 5f and h, lanes 1 & 3). Iron overloading resulted in the downregulation of GLUT1 and GLUT2 in Tg40 PrP mice, but not in PrP^−/−^ mice (Fig. 5f and h, lanes 2 & 4; Fig. 5g and i).

All the above investigation was also carried out in the C6 mice in both the strains; C6 PrP^+/+^ and C6 PrP^−/−^, with similar results in all 4 tissues (see Supplementary Fig. S5).

### PrP-mediated increase in β-cell iron downregulates insulin

**Figure 6:**
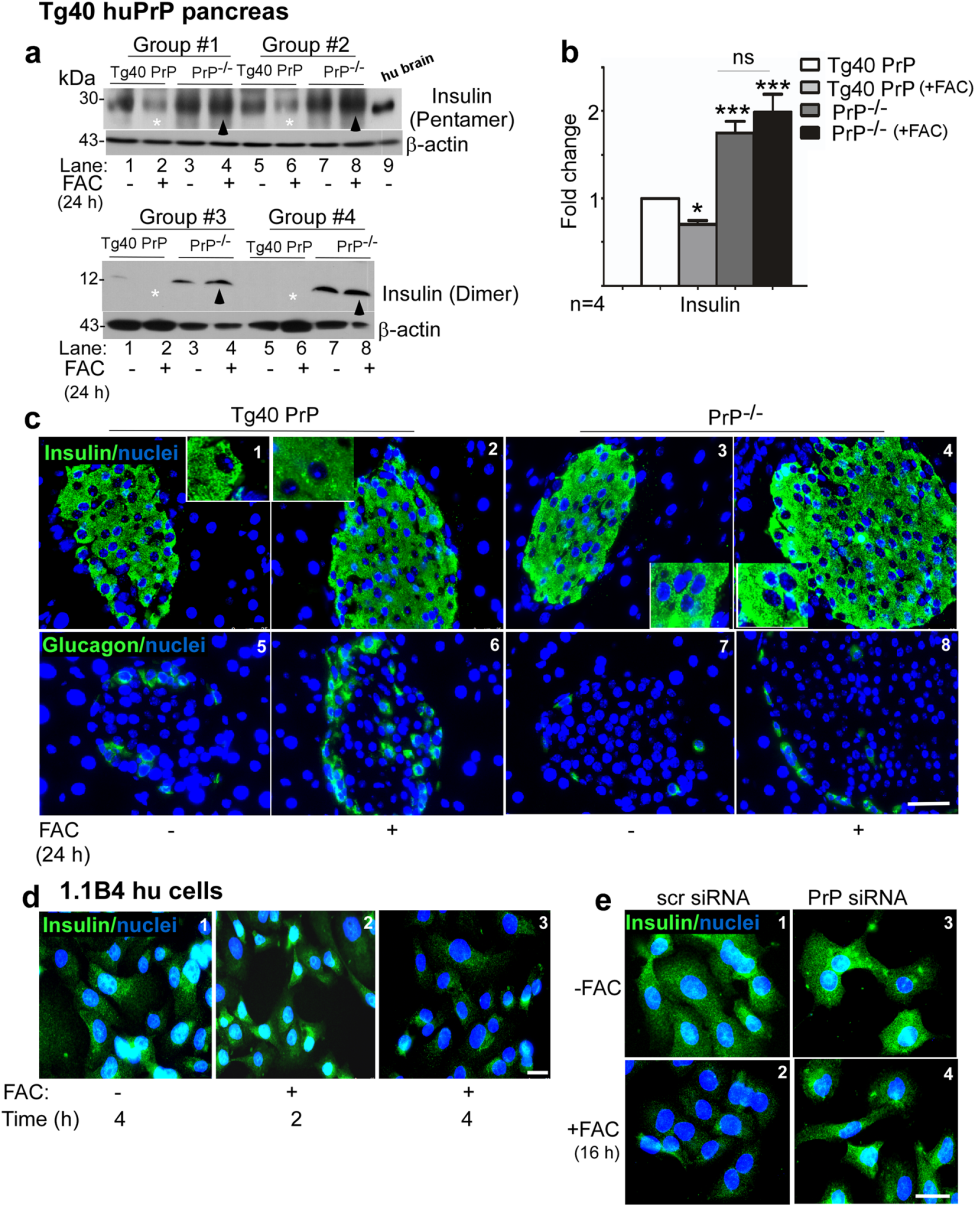
PrP-mediated increase in β-cell iron decreases insulin: **(a)** Probing of Western blots of pancreatic lysates from control and iron overloaded Tg40 PrP and PrP^−/−^ mice with antibodies specific for insulin dimer and pentamer (upper and lower panels) shows decreased expression in Tg40 PrP relative to PrP^−/−^ samples (lanes 1 & 5 vs. 3 & 7; **b**). Iron overloading decreases insulin levels in Tg40 PrP (lanes 1vs. 2 & 5 vs. 6; **b**), but has minimal effect on PrP^−/−^ samples (lanes 3 vs. 4 & 7 vs. 8; **b**). **(b)** Density of protein bands after normalization with β-actin. Values are mean ± SEM of the indicated n. *p < 0.05, ***p < 0.001, ns, not significant. The full-length blots are provided in Supplementary File (Raw data). **(c)** Immunohistochemistry of pancreas shows relatively higher reactivity for insulin in PrP^−/−^ relative to Tg40 PrP sections (panels 1 & 3). Iron overloading decreases insulin reactivity in Tg40 PrP but not in PrP^−/−^ samples (panels 1 vs. 2 & 3 vs. 4). Reaction for glucagon is higher in iron overloaded Tg40 PrP relative to control samples (panels 5 & 6). The difference is minimal in PrP^−/−^ samples (panels 7 & 8). **(d)** Immunostaining of 1.1B4 cells for insulin shows decreased reactivity after 2 and 4 hours of exposure to FAC (panels 1–3). **(e)** Silencing of PrP with siRNA abrogates iron-mediated decrease in insulin reactivity (panels 1–4). Scale bar 20 μm.

To evaluate whether expression of GLUT2 influences insulin levels in β-cells, pancreatic lysates from control and iron overloaded Tg40 PrP mice were subjected to Western blotting and probed for insulin with two different antibodies^43,44^. Essentially the same observations were noted as for GLUT2 (Fig. 4). Reactivity for insulin was significantly higher in PrP^−/−^ relative to Tg40 PrP samples. Iron overloading decreased insulin in Tg40 PrP, but had minimal effect on PrP^−/−^ samples (Fig. 6a, lanes 1–8, upper and lower panels; Fig. 6b). This experiment was also conducted in C6 mice in both the strains; C6 PrP^+/+^ and C6 PrP^−/−^, with similar results (see Supplementary Fig. S6).

Immunoreaction of fixed pancreas for insulin showed reactivity on β-cells as expected. The intensity of insulin reactivity was higher in PrP^−/−^ relative to Tg40 PrP samples, and excess iron reduced the reaction in Tg40 PrP but had minimal effect on PrP^−/−^ samples, consistent with Western blot results in panel A (Fig. 6c, panels 1–4). A similar analysis for glucagon showed increased reactivity in iron overloaded Tg40 PrP samples, indicating proliferation of glucagon producing α-cells by this treatment^45^. PrP^−/−^ samples, on the other hand, showed minimal reactivity in untreated and iron overloaded samples (Fig. 6c, panels 5–8).

To confirm a causal relationship between β-cell iron levels and insulin, 1.1B4 cells were exposed to FAC for 2 and 4 hours, and immunostained for insulin. Reactivity for insulin decreased after 2 h, and was negligible after 4 h. Nuclear staining revealed normal nuclear morphology with no obvious signs of toxicity (Fig. 6d, panels 1–3). Downregulation of PrP^C^ by siRNA, however, reversed the negative effect of iron on insulin reactivity (Fig. 6e, panels 1–4).

Together, the above observations demonstrate that increase in β-cell iron results in downregulation of GLUT2 and insulin, and PrP^C^ plays a significant role in this process by facilitating iron uptake.

### PrP^C^-mediated dysregulation of blood glucose is exacerbated by iron

**Figure 7:**
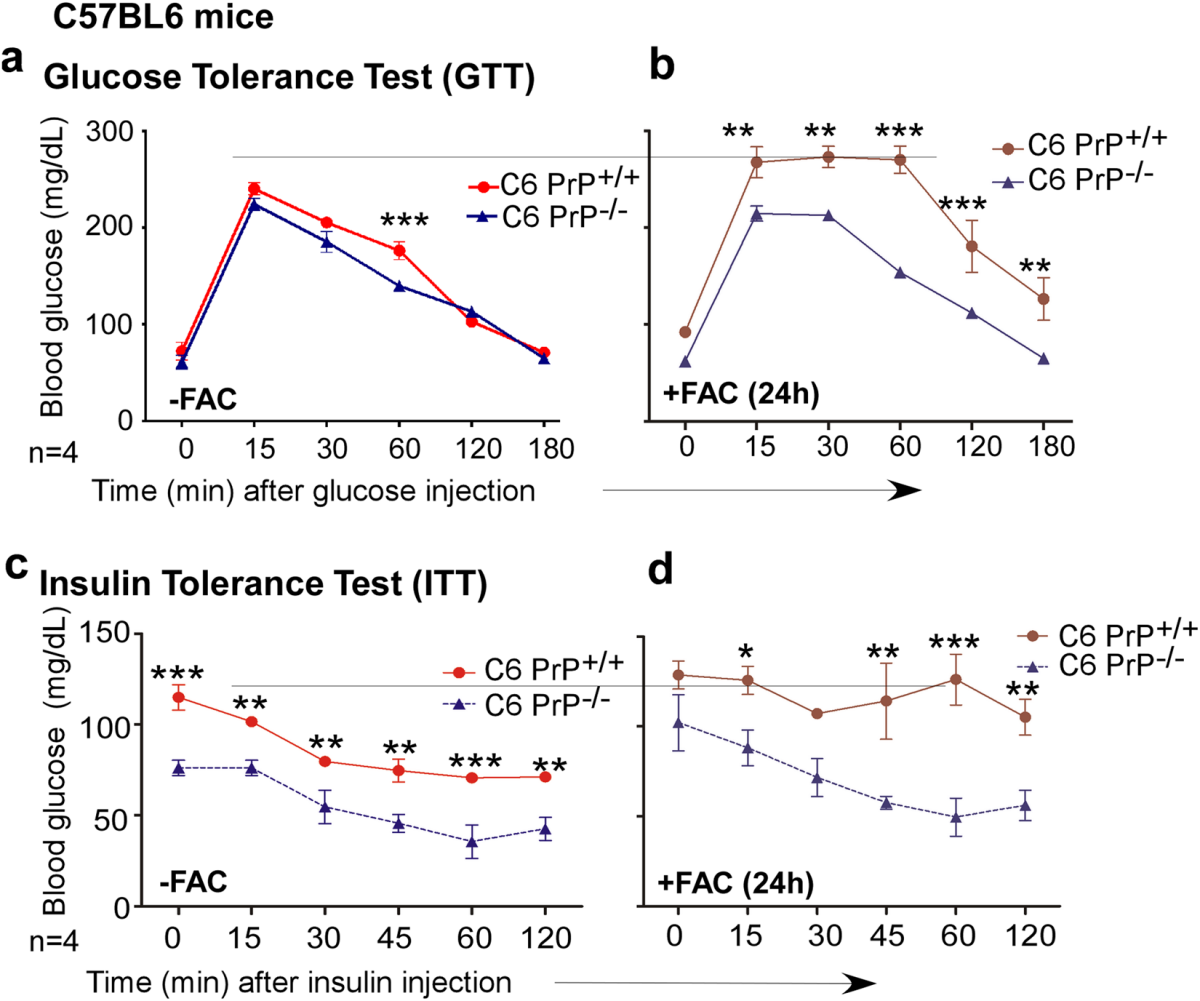
PrP-mediated increase in IC iron blunts the systemic response to peripheral challenge with glucose and insulin: (**a)** GTT in C6 PrP^+/+^ and C6 PrP^−/−^ mice shows an initial spike in blood glucose in both mouse lines after 15 min, followed by a gradual decline to control levels by 180 min. Blood glucose is significantly higher in C6 PrP^+/+^ relative to C6 PrP^−/−^ mice at the 60 min time point. **(b)** Iron overloading decreases glucose tolerance in C6 PrP^+/+^ relative to C6 PrP^−/−^ mice at all-time points tested. **(c)** ITT shows a hypoglycemic response in C6 PrP^−/−^ mice relative to that C6 PrP^+/+^ controls. **(d)** Iron overloading increases insulin resistance in C6 PrP^+/+^ mice, but has minimal effect on C6 PrP^−/−^ mice. The data represent mean ± SEM of values from two different strains of mice. *p < 0.05, **p < 0.01, ***p < 0.001.

To evaluate whether decreased reactivity for insulin in PrP^C^-expressing β-cells and exaggeration of this phenotype by excess iron is due to decreased synthesis or rapid release from cells, control and iron overloaded C6 PrP^+/+^ and C6 PrP^−/−^ mice were injected with glucose or insulin according to guidelines for conducting glucose tolerance test (GTT) and insulin tolerance test (ITT) in laboratory mice^46^. Blood glucose was measured and recorded at the indicated times (Fig. 7). As expected, both mouse lines showed a spike in blood glucose after 15 min (T-15), and a gradual decline to basal levels (T-0) after 180 min (Fig. 7a). It is notable that blood glucose was significantly higher in C6 PrP^+/+^ samples at T-60 relative to C6 PrP^−/−^ samples (Fig. 7a). This difference was more widespread and exaggerated following over-loading, and the blood glucose of C6 PrP^+/+^ mice was significantly higher than C6 PrP^−/−^ at all the time-points tested (Fig. 7b). The results from ITT were more dramatic. Following an injection of insulin, the blood in C6 PrP^+/+^ mice remained significantly higher than C6 PrP^−/−^ samples at all the time points tested regardless of iron overloading (Fig. 7c & d).

Relatively poor glucose tolerance in C6 PrP^+/+^ mice especially after iron overloading suggests reduced levels of circulating insulin in response to glucose, consistent with decreased levels of GLUT2 and insulin in pancreatic β-cells observed above. Impaired response of C6 PrP^+/+^ mice to injected insulin regardless of iron overload suggests peripheral resistance to insulin that increases after iron overload.

Together, the above observations indicate that C6 PrP^+/+^ mice display a phenotype of type-2-diabetes, i.e. impaired synthesis of insulin and peripheral resistance to available insulin^47^, exacerbated further by iron overload. C6 PrP^−/−^ mice, on the other hand, are relatively resistant to iron-mediated fluctuations in blood glucose. These experiments were also conducted on Tg40 PrP and PrP^−/−^ mice with similar trend in GTT and ITT results.

## Discussion

**Figure 8:**
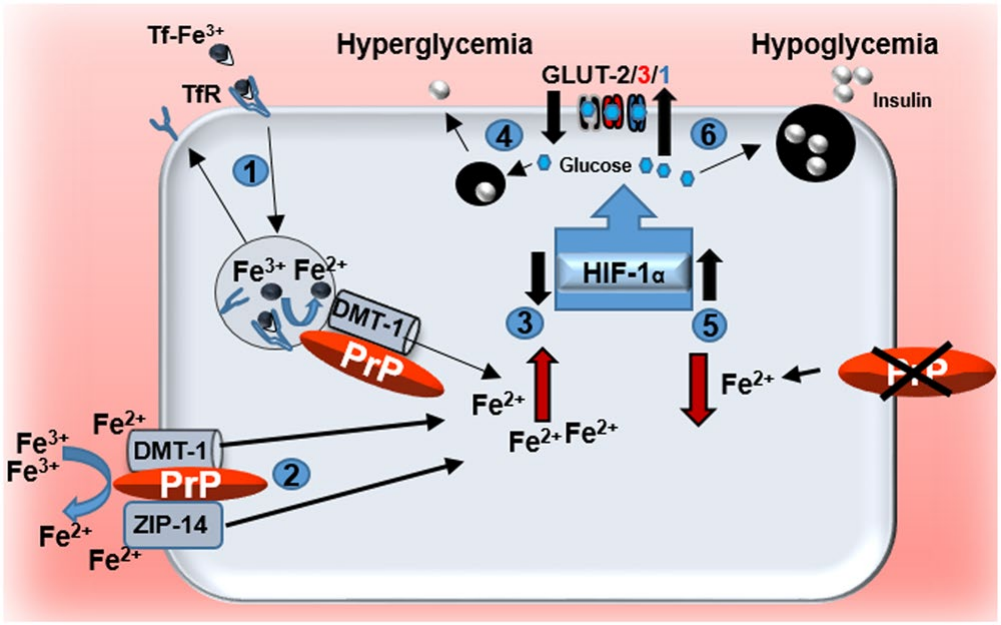
Graphical representation of PrP^C^-mediated modulation of glucose homeostasis through iron. **(1 & 2)** PrP^C^ facilitates cellular uptake of Tf-Fe^3+^ and non-Tf-bound iron (Fe^3+^) by functioning as a ferrireductase partner for DMT-1 and ZIP14^20^. **(3)** The increase in intracellular (IC) iron down-regulates HIF1α^53^, resulting in the downregulation of GLUT2 in pancreatic β-cells and hepatocytes (black), GLUT 3 in neuronal cells (red), and GLUT1 at the blood-retinal barrier (blue). **(4)** Reduced uptake of glucose downregulates insulin, resulting in hyperglycemia. **(5)** Down-regulation or deletion of PrP^C^ decreases IC iron, resulting in the upregulation of HIF1α^53^ and glucose transporters. **(6)** Increased uptake of glucose stimulates insulin secretion with resultant hypoglycemia.

Our data demonstrate a direct correlation between PrP^C^ and IC iron, and a converse relationship between IC iron and glucose uptake in pancreatic β-cells, neuronal cells, hepatocytes, and the retina. In pancreatic β-cells, PrP^C^-mediated increase in IC iron and downregulation of GLUT2 reduced the synthesis and release of insulin. In peripheral organs, iron overloading increased peripheral resistance to insulin in a PrP^C^-dependent manner, suggesting a dual role of PrP^C^-mediated increase in systemic iron on glucose homeostasis. Experimental conditions show that stimulated iron uptake by the pancreas resulted in β-cleavage of PrP^C^, indicating regulation of iron uptake through this process. Together, these observations explain the mechanism underlying PrP^C^-mediated modulation of blood glucose^17^, and confirm the positive correlation between iron and type-2-diabetes^2^ (Fig. 8). Since GLUT3 is downregulated in AD and prion disease affected brains^48,49^, conditions associated with brain iron dyshomeostasis^50^, it is likely that PrP^C^ induces toxicity by the combined effect of oxidative stress and glucose deprivation^51^.

It was surprising that PrP^C^ had a significant effect on IC iron in pancreatic β-cells despite relatively low expression^17^. PrP^C^ was localized to insulin-producing β-cells in mouse pancreas, and showed the expected glycoforms as in neuronal cells. However, unlike neuronal cells, most of the PrP^C^ on pancreatic β-cells and 1.1B4 cells was cleaved at the β-site, a processing event that was triggered by exogenous iron and over-expression of divalent metal transporters DMT-1 and ZIP14. The concomitant increase in cellular ferritin suggested that β-cleavage of PrP^C^ is coupled with iron uptake^32^, and proteolytic cleavage of PrP^C^ could serve to regulate iron uptake^35^. Further exploration is necessary to understand this phenomenon. The expression and functional activity of ZIP14 and DMT-1 on human and mouse pancreatic β-cells has been described recently^38,52^, and supports the above assumption.

A positive correlation between systemic iron and type-2-diabetes is well-established, and several studies have addressed the underlying mechanism^2,4^. The increase in intracellular (IC) iron down-regulates hypoxia inducible factors 1 (HIF1α)^53^, resulting in the downregulation of GLUTs. The inverse scenario where hyperglycemia results in iron dysregulation has also been studied^54^. Our data strengthen these observations, and provide a novel mechanism of glucose modulation through PrP^C^, a mainly neuronal protein, thereby linking both peripheral and neuronal glucose homeostasis to IC iron. Given the relatively low expression of PrP^C^ on pancreatic β-cells in comparison to neurons, reduced expression of GLUT2 and insulin in the pancreas of Tg40 PrP relative to PrP^−/−^ mice was intriguing, and suggested that even minor changes in β-cell IC iron are sufficient to alter glucose homeostasis. Systemic iron overload amplified this effect and increased peripheral resistance to insulin^47^, simulating the phenotype of type-2-diabetes. PrP^−/−^ mice, on the other hand, remained unaffected, demonstrating a direct correlation between iron, GLUT2, and insulin. The dose of iron used in our study was within the recommended range for the treatment of anemia, making it unlikely that the observed effects are due to iron-induced β-cell toxicity^4^. Knock-out of ZIP14 in mouse pancreas has been reported to induce hyperinsulinemia and hypoglycemia due to iron deficiency as in PrP^−/−^ mice, lending support to our observations^55^. Similar observations were noted in the liver of Tg40 PrP and PrP^−/−^ mice, indicating that the inverse correlation between IC iron and glucose transport is not limited to the pancreas, and relatively low expression of PrP^C^ is sufficient to induce this change. Our results differ from a previous report indicating impaired or delayed response of PrP^−/−^ mice to hyperglycemia^17^, a discrepancy that is difficult to explain from our data. However, diverse observations regarding PrP^C^ and glucose homeostasis have been reported in the literature^56–62^ requiring further exploration on this subject.

Reduced expression of GLUT3 in brain homogenates and GLUT1 in the neuroretina of Tg40 PrP relative to PrP^−/−^ suggests similar regulation of IC iron and glucose transporters by PrP^C^ as in pancreatic β-cells. These observations have significant implications for neuronal cells that express high levels of PrP^C^ and require glucose for their high metabolic demands. Since several neurodegenerative diseases including AD and Creutzfeldt–Jakob disease (sCJD) are associated with neuronal iron dyshomeostasis^12^, it is likely that in pathological conditions such as these, PrP^C^ accentuates neuronal injury by the combined effect of iron-mediated oxidative stress and glucose deprivation. It is notable that GLUT3 is downregulated in scrapie infected animal brains^49^ and human cases of CJD^63^. A similar downregulation of GLUT3 has been observed in AD brains^48^, a known long-term complication of type-2-diabetes^10^. Whether PrP^C^ is the principal regulator of GLUT3 expression in neurons is not clear from our data, and remains an open question. However, considering our observations on pancreatic β-cells and the similarities between GLUT2 and GLUT3^21^, it is tempting to speculate that PrP^C^ plays a similar role in neurons, and is likely to influence neuronal health under normal and pathological conditions by modulating iron and glucose uptake^24^. Likewise, PrP^C^ is expressed widely in the neuroretina, including retinal pigment epithelial cells in the outer blood retinal barrier where it mediates uptake of iron by the neuroretina. PrP^C^ is therefore likely to modulate both iron and glucose homeostasis in the retina, that, like the brain, has a high metabolic rate and depends on glucose for normal function.

In conclusion, our observations reveal a novel function of PrP^C^ in regulating blood glucose through iron, and reaffirm the correlation between systemic iron and type-2-diabetes. The relatively high expression of PrP^C^ in the brain and the neuroretina underscores its significance as a key protein that regulates iron and glucose homeostasis, vital processes critical for the functioning of these metabolically active tissues. The correlation between PrP^C^, iron, and glucose homeostasis is likely to provide new, untapped therapeutic opportunities for type-2-diabetes, AD, and other neurodegenerative conditions associated with iron dyshomeostasis.

## Materials and Methods

### Ethics statement

All animals were housed in the Association for Assessment and Accreditation of Laboratory Animal Care International (AAALAC)-approved Animal Resource Center (ARC) at Case Western Reserve University (CWRU) School of Medicine (SOM) under a 12h day-night cycle, and provided ad libitum access to food and water. All experimental procedures were reviewed and approved by the CWRU IACUC committee in accordance with provisions of the Animal Welfare Act and Guide for the Care and Use and of Laboratory Animals as well as the U.S. Government Principles for the Utilization and Care of Vertebrate Animals Used in Testing, Research, and Training (animal protocol # 2015–0027). The ARC at CWRU is directed by Dr. Durfee, DVM, Diplomate ACLAM, and daily animal care is provided by two full-time veterinarians. The CWRU PHS Assurance number A-3145–01 is valid until 04/30/19.

### Mouse strains

Four mouse strains were used to improve the validity of this study; PrP-knock out (PrP^−/−^) mice deposited by^25^ to Jackson Laboratories (cat # 129-Prnptm2Edin/J Stock No: 012938) and crossed with C57BL/6 wild-type mice for 10 generations. F2 generation of wild type (C6 PrP^+/+^) and corresponding PrP^−/−^ (C6 PrP^−/−^) were used for these studies. FVB/NJ Tg40 (Tg40 PrP) that express 2 × human PrP were generated from FVB/Prnp^00^ mice^26^ kindly provided by the Prusiner laboratory and used as corresponding controls for Tg40 PrP mice^64^. All mouse lines were fed regular chow (Prolab Isopro RMH 3000 from www.labdiet.com) and maintained under similar conditions. All experiments were conducted on 6–8 week old male mice since females show less pronounced phenotype of glucose intolerance^17^, and carried out at the same time of the day.

### Chemicals

Alexa Fluor 546-tagged secondary antibodies (A11071, A11018) were from Southern Biotech, USA and Molecular Probes, USA respectively. PNGase F (P0704S) was from New England Biolabs (NEB), USA, Lipofectamine 3000 was from Invitrogen, USA. Ferric ammonium citrate (FAC) (F5879) and desferrioxamine (D9533) were from Sigma Aldrich, USA, D-glucose (dextrose-15023021) was from ThermoFisher Scientific, USA, and Humulin-R was from Eli Lilly, USA. siRNA against PrP (sc36318) and scrambled siRNA (sc37007) were from Santa Cruz Biotechnology Inc, USA.

Scrambled siRNA sequence: Sense: UUCUCCGAACGUGUCACGUtt

Antisense: ACGUGACACGUUCGGAGAAtt

PrP siRNA (h) is a pool of 3 different siRNA duplexes: sc-36318A: Sense: GUGACUAUGAGGACCGUUAtt

Antisense: UAACGGUCCUCAUAGUCACtt sc-36318B: Sense: GAGACCGACGUUAAGAUGAtt

Antisense: UCAUCUUAACGUCGGUCUCtt sc-36318C: Sense: GUUGAGCAGAUGUGUAUCAtt

Antisense: UGAUACACAUCUGCUCAACtt

Note: all sequences are in 5′ → 3′ orientation.

Plasmid encoding DMT-1-GFP (-IRE) was from Jerry Kaplan (University of Utah). PrP^C^ and PrP^C^-GFP constructs were prepared by ligating cDNA from previously reported constructs in modified PiggyBac vectors (System Biosciences, Mountain view, CA)^34^. Plasmids encoding ZIP14 and ZIP8 tagged with GFP were obtained from Mitchell D. Knutson, PhD (University of Florida)^65^. Transfection was carried out using Lipofectamine3000 transfection kit (Invitrogen, USA).

### Cell lines, transfection, and RNAi knockdown

Insulin producing human pancreatic β-cell line 1.1B4 was obtained from Sigma Aldrich (Cat. No: 10012801), USA and cultured as described^66^. This is a PANC-1 hybrid human pancreatic beta cell line that secretes insulin. Cells were cultured in RPMI-1640 supplemented with 2 mM Glutamine and 10% heat inactivated FBS. Cells were passaged every fourth day. For silencing PrP^C^, 1.1B4 cells were transfected with siRNA against PrP^C^ or scrambled control using Lipofectamine RNAimax (Invitrogen). After 72 h, cells were exposed to 16.7 mM of glucose for 1 h and processed for Western blotting. Human neuroblastoma cells (M17) were purchased from ATCC. Cells expressing vector or PrP^C^ were generated described in previous reports^67,68^, and cultured in DMEM supplemented with 10% FBS. All cell lines were maintained at 37 °C in a humidified atmosphere containing 5% CO_2_.

### Antibodies

PrP^C^-specific antibodies 3F4 and 8H4 were from Signet laboratories (Dedham, MA) and Sigma Aldrich respectively. Other antibodies were obtained from the following sources: ferritin specific for heavy and light chain (F5012) from Sigma Aldrich, USA, GLUT-1 (NB110-39113) from Novus Biologicals, GLUT-2 (ab54460), and GLUT-3 (ab41525) from Abcam, USA, TfR (13-6800) from Invitrogen, USA, insulin from Santa Cruz Biotechnology Inc. (sc-9168) and Novus Biologicals (NBP2-34260), USA (recognize insulin and a 51-amino acid polypeptide composed of A and B chains connected through the C-peptide), glucagon (sc-13091) from Santa Cruz Biotechnology Inc, USA, and β-actin (MAB1501) from Millipore, USA. HRP-conjugated secondary antibodies (anti-mouse, NA931V, anti-rabbit, NA934V) were from GE Healthcare, UK.

### Iron treatment

1.1B4 and M17 cells cultured in complete medium were exposed to vehicle or 30 μM of ferric ammonium citrate (FAC) for 16 h at 37 °C before processing for immunostaining and Western blot as described^69^. To create systemic iron overload, age and sex-matched C6 PrP^+/+^, C6 PrP^−/−^, Tg40 PrP and PrP^−/−^ mice were injected intraperitoneally with 36 µg/22 g mouse weight of FAC and euthanized after 24 h for further analysis^37^. The dose of iron used (1.6 mg/kg body weight) is well below the recommended ~10 mg/kg/day used for the treatment of anemia in an average adult weighing 75 kg^70^.

### Western blotting and Immunostaining

Western blotting and immunostaining were carried out as described^69^. In short, cells and tissues were lysed in RIPA lysis buffer (50 mM Tris-HCl pH 7.4, 100 mM NaCl, 1% NP-40, 0.5% deoxycholate), boiled in reducing gel-loading buffer for 5 min at 100 °C, and fractionated by SDS-PAGE. Fractionated proteins were transferred to PVDF membranes and probed for specific proteins. Quantification of protein bands was performed by densitometry using UN-SCAN-IT gels (version6.1) software (Silk Scientific) and analyzed graphically using GraphPad Prism (Version 5.0) software (GraphPad Software Inc.).

### Glucose Tolerance Test (GTT) and Insulin Tolerance Test (ITT)

GTT and ITT were performed in age and sex-matched mice at the same time of day as described^46^. For GTT, the mice were fasted overnight with *ad libidum* access to water, and 1 g glucose/kg body weight was injected intraperitoneally. Blood glucose was monitored at 0, 15, 30, 60, 120, and 180 min post-injection with a glucometer (EasyMax-Diabetic Promotions, USA). For ITT, the mice had *ad libidum* access to food and water because PrP^−/−^ mice went into hypoglycemic shock after insulin injection. Accordingly, non-fasted animals were injected with 0.75 U insulin/kg body weight intraperitoneally, and blood glucose was monitored as above at 0, 15, 30, 45, 60 and 120 min post-injection.

### Statistical analysis

Data were analyzed using GraphPad Prism5 (GraphPad Software, Inc., La Jolla, CA) and presented as Mean ± SEM. Level of significance was calculated by Two-way ANOVA between the control and experimental groups.

## Electronic supplementary material

Supplementary information

## References

[1] Skyler JS (2017). Differentiation of diabetes by pathophysiology, natural history, and prognosis. Diabetes.

[2] Simcox JA, McClain DA (2013). Iron and diabetes risk. Cell metabolism.

[3] Swaminathan S, Fonseca VA, Alam MG, Shah SV (2007). The Role of Iron in Diabetes and Its Complications. Diabetes Care.

[4] Rajpathak SN (2009). The role of iron in type 2 diabetes in humans. Biochimica et Biophysica Acta (BBA)-General Subjects.

[5] Fernandez-Real JM, Lopez-Bermejo A, Ricart W (2005). Iron stores, blood donation, and insulin sensitivity and secretion. Clin Chem.

[6] Cario H, Holl RW, Debatin KM, Kohne E (2003). Insulin sensitivity and beta-cell secretion in thalassaemia major with secondary haemochromatosis: assessment by oral glucose tolerance test. Eur J Pediatr.

[7] McClain DA (2006). High prevalence of abnormal glucose homeostasis secondary to decreased insulin secretion in individuals with hereditary haemochromatosis. Diabetologia.

[8] Acton RT (2006). Relationships of serum ferritin, transferrin saturation, and HFE mutations and self-reported diabetes in the Hemochromatosis and Iron Overload Screening (HEIRS) study. Diabetes Care.

[9] Lee DH, Folsom AR, Jacobs DR (2004). Dietary iron intake and Type 2 diabetes incidence in postmenopausal women: the Iowa Women’s Health Study. Diabetologia.

[10] Cheng D (2011). Type 2 diabetes and late-onset Alzheimer’s disease. Dementia and geriatric cognitive disorders.

[11] Correia SC (2012). Insulin signaling, glucose metabolism and mitochondria: Major players in Alzheimer’s disease and diabetes interrelation. Brain Research.

[12] Belaidi AA, Bush AI (2016). Iron neurochemistry in Alzheimer’s disease and Parkinson’s disease: targets for therapeutics. Journal of neurochemistry.

[13] Ciudin, A., Hernández, C. & Simó, R. Iron overload in diabetic retinopathy: a cause or a consequence of impaired mechanisms? *Experimental diabetes research* **2010** (2010). 10.1155/2010/714108 PMC2935195 20827392

[14] Prusiner, S. B. P. Proceedings of the National Academy of Sciences **95**, 13363–13383 (1998). 10.1073/pnas.95.23.13363 PMC33918 9811807

[15] Wulf M-A, Senatore A, Aguzzi A (2017). The biological function of the cellular prion protein: an update. BMC biology.

[16] Gains MJ, Roth KA, LeBlanc AC (2006). Prion protein protects against ethanol-induced Bax-mediated cell death in vivo. Neuroreport.

[17] Strom A, Wang G-S, Scott FW (2011). Impaired glucose tolerance in mice lacking cellular prion protein. Pancreas.

[18] Strom A, Wang GS, Reimer R, Finegood DT, Scott FW (2007). Pronounced cytosolic aggregation of cellular prion protein in pancreatic beta-cells in response to hyperglycemia. Lab Invest.

[19] de Brito G (2017). Loss of prion protein is associated with the development of insulin resistance and obesity. Biochem J.

[20] Tripathi AK (2015). Prion protein functions as a ferrireductase partner for ZIP14 and DMT1. Free Radical Biology and Medicine.

[21] Mueckler M, Thorens B (2013). The SLC2 (GLUT) family of membrane transporters. Molecular aspects of medicine.

[22] Thorens B (2015). GLUT2, glucose sensing and glucose homeostasis. Diabetologia.

[23] Sansbury F (2012). SLC2A2 mutations can cause neonatal diabetes, suggesting GLUT2 may have a role in human insulin secretion. Diabetologia.

[24] Singh N (2014). Iron in neurodegenerative disorders of protein misfolding: a case of prion disorders and Parkinson’s disease. Antioxidants & redox signaling.

[25] Manson JC (1994). 129/Ola mice carrying a null mutation in PrP that abolishes mRNA production are developmentally normal. Molecular neurobiology.

[26] Fischer M (1996). Prion protein (PrP) with amino-proximal deletions restoring susceptibility of PrP knockout mice to scrapie. The EMBO journal.

[27] Chen SG (1995). Truncated forms of the human prion protein in normal brain and in prion diseases. The Journal of biological chemistry.

[28] Singh A (2009). Prion Protein Modulates Cellular Iron Uptake: A Novel Function with Implications for Prion Disease Pathogenesis. PLOS ONE.

[29] Tank EM, Harris DA, Desai AA, True HL (2007). Prion protein repeat expansion results in increased aggregation and reveals phenotypic variability. Molecular and cellular biology.

[30] Ma J, Wang F (2014). Prion disease and the ‘protein-only hypothesis’. Essays in biochemistry.

[31] Masujin K (2016). Detection of atypical H-type bovine spongiform encephalopathy and discrimination of bovine prion strains by real-time quaking-induced conversion. Journal of clinical microbiology.

[32] Watt NT (2005). Reactive oxygen species-mediated β-cleavage of the prion protein in the cellular response to oxidative stress. Journal of Biological Chemistry.

[33] Mangé A (2004). Alpha‐and beta‐cleavages of the amino‐terminus of the cellular prion protein. Biology of the Cell.

[34] Gu Y (2003). Mutant prion protein-mediated aggregation of normal prion protein in the endoplasmic reticulum: implications for prion propagation and neurotoxicity. J Neurochem.

[35] Asthana A (2017). Prion protein facilitates retinal iron uptake and is cleaved at the β-site: Implications for retinal iron homeostasis in prion disorders. Scientific Reports.

[36] Arredondo M, Mendiburo MJ, Flores S, Singleton ST, Garrick MD (2014). Mouse divalent metal transporter 1 is a copper transporter in HEK293 cells. BioMetals.

[37] Haldar S (2015). Prion protein promotes kidney iron uptake via its ferrireductase activity. The Journal of biological chemistry.

[38] Coffey R, Knutson MD (2017). The plasma membrane metal-ion transporter ZIP14 contributes to nontransferrin-bound iron uptake by human β-cells. American Journal of Physiology - Cell Physiology.

[39] Singh A (2009). Prion protein (PrP) knock-out mice show altered iron metabolism: a functional role for PrP in iron uptake and transport. PLoS One.

[40] Pushie MJ (2011). Prion protein expression level alters regional copper, iron and zinc content in the mouse brain. Metallomics.

[41] Singh A (2013). Prion protein regulates iron transport by functioning as a ferrireductase. Journal of Alzheimer’s Disease.

[42] Simpson IA (2008). The facilitative glucose transporter GLUT3: 20 years of distinction. American Journal of Physiology-Endocrinology and Metabolism.

[43] Chakraborty D, Samadder A, Dutta S, Khuda-Bukhsh AR (2012). Antihyperglycemic potentials of a threatened plant, Helonias dioica: antioxidative stress responses and the signaling cascade. Experimental Biology and Medicine.

[44] Dosanjh, J. K. *Protein damage during purification: understanding the effects of size exclusion chromatography on the structure of biosynthetic human insulin (BHI)*, UCL (University College London), (2011).

[45] Takeda Y (2012). Reduction of both beta cell death and alpha cell proliferation by dipeptidyl peptidase-4 inhibition in a streptozotocin-induced model of diabetes in mice. Diabetologia.

[46] Ayala JE (2010). Standard operating procedures for describing and performing metabolic tests of glucose homeostasis in mice. Disease Models & Mechanisms.

[47] Chatterjee S, Khunti K, Davies MJ (2017). Type 2 diabetes. The Lancet.

[48] Simpson IA, Chundu KR, Davies‐Hill T, Honer WG, Davies P (1994). Decreased concentrations of GLUT1 and GLUT3 glucose transporters in the brains of patients with Alzheimer’s disease. Annals of neurology.

[49] Yan YE (2014). Significant reduction of the GLUT3 level, but not GLUT1 level, was observed in the brain tissues of several scrapie experimental animals and scrapie-infected cell lines. Molecular neurobiology.

[50] Blasco G (2014). Brain iron overload, insulin resistance, and cognitive performance in obese subjects: a preliminary MRI case-control study. Diabetes Care.

[51] Singh N (2014). The role of iron in prion disease and other neurodegenerative diseases. PLoS pathogens.

[52] Hansen JB (2012). Divalent metal transporter 1 regulates iron-mediated ROS and pancreatic β cell fate in response to cytokines. Cell metabolism.

[53] Cheng K (2010). Hypoxia-inducible factor-1alpha regulates beta cell function in mouse and human islets. J Clin Invest.

[54] Rowe PA, Kavanagh K, Zhang L, Harwood HJ, Wagner JD (2011). Short-term hyperglycemia increases arterial superoxide production and iron dysregulation in atherosclerotic monkeys. Metabolism.

[55] Aydemir TB (2012). Zinc transporter ZIP14 functions in hepatic zinc, iron and glucose homeostasis during the innate immune response (endotoxemia). PLoS One.

[56] Li QQ (2011). Cellular prion protein promotes glucose uptake through the Fyn-HIF-2alpha-Glut1 pathway to support colorectal cancer cell survival. Cancer science.

[57] Kuwahara C (2000). Enhanced expression of cellular prion protein gene by insulin or nerve growth factor in immortalized mouse neuronal precursor cell lines. Biochem Biophys Res Commun.

[58] Bitel CL, Feng Y, Souayah N, Frederikse PH (2010). Increased expression and local accumulation of the prion protein, Alzheimer Aβ peptides, superoxide dismutase 1, and nitric oxide synthases 1 & 2 in muscle in a rabbit model of diabetes. BMC physiology.

[59] Pham N, Dhar A, Khalaj S, Desai K, Taghibiglou C (2014). Down regulation of brain cellular prion protein in an animal model of insulin resistance: possible implication in increased prevalence of stroke in pre-diabetics/diabetics. Biochemical and biophysical research communications.

[60] Zhu C, Schwarz P, Abakumova I, Aguzzi A (2015). Unaltered prion pathogenesis in a mouse model of high-fat diet-induced insulin resistance. PloS one.

[61] Nielsen D, Gyllberg H, Ostlund P, Bergman T, Bedecs K (2004). Increased levels of insulin and insulin-like growth factor-1 hybrid receptors and decreased glycosylation of the insulin receptor alpha-and beta-subunits in scrapie-infected neuroblastoma N2a cells. Biochemical Journal.

[62] Östlund P, Lindegren H, Pettersson C, Bedecs K (2001). Altered insulin receptor processing and function in scrapie-infected neuroblastoma cell lines. Molecular Brain Research.

[63] Kim EJ (2012). Glucose metabolism in sporadic Creutzfeldt–Jakob disease: a statistical parametric mapping analysis of 18F‐FDG PET. European journal of neurology.

[64] Kong Q (2005). Chronic Wasting Disease of Elk: Transmissibility to Humans Examined by Transgenic Mouse Models. The Journal of Neuroscience.

[65] Coffey R, Knutson MD (2017). The plasma membrane metal-ion transporter ZIP14 contributes to nontransferrin-bound iron uptake by human β-cells. American Journal of Physiology-Cell Physiology.

[66] Vasu S, McClenaghan NH, McCluskey JT, Flatt PR (2014). Mechanisms of toxicity by proinflammatory cytokines in a novel human pancreatic beta cell line, 1.1B4. Biochimica et Biophysica Acta (BBA) - General Subjects.

[67] Gu Y, Fujioka H, Mishra RS, Li R, Singh N (2002). Prion peptide 106-126 modulates the aggregation of cellular prion protein and induces the synthesis of potentially neurotoxic transmembrane PrP. The Journal of biological chemistry.

[68] Jin T (2000). The chaperone protein BiP binds to a mutant prion protein and mediates its degradation by the proteasome. The Journal of biological chemistry.

[69] Baksi S, Tripathi AK, Singh N (2016). Alpha-synuclein modulates retinal iron homeostasis by facilitating the uptake of transferrin-bound iron: Implications for visual manifestations of Parkinson’s disease. Free Radical Biology and Medicine.

[70] Koch, T. A., Myers, J. & Goodnough, L. T. Intravenous iron therapy in patients with iron deficiency anemia: dosing considerations. *Anemia* **2015** (2015). 10.1155/2015/763576 PMC4518169 26257955

