# Supplementary material for: Prion protein modulates glucose homeostasis by altering intracellular iron
Source: Sci Rep. 2018 Apr 26;8:6556. doi: 10.1038/s41598-018-24786-1 (PMC5919926; doi:10.1038/s41598-018-24786-1)
Supplement: Supplementary file 1 — Supplementary information [file 41598_2018_24786_MOESM1_ESM.pdf]

Supplementary Data

Prion protein modulates glucose homeostasis by altering intracellular iron

Ajay Ashok, Neena Singh*

***Corresponding author**
E-mail: neena.singh@case.edu
Tel: 216-368-2617

Department of Pathology, School of Medicine, Case Western Reserve University, Cleveland, Ohio 44106, USA.

Supplementary Fig S1: Uptake of NTBI by the pancreas

Next to the liver, pancreas, heart, and the kidney take up more NTBI relative to Tf-Fe, while the spleen and bone marrow incorporate Tf-Fe preferentially (Craven, 1978)[1]. To confirm this phenomenon in $PrP^{+/+}$ and $PrP^{-/-}$ mice used in this study, mice were injected with unlabeled ferric ammonium citrate (FAC) to saturate plasma Tf, followed by equal counts of ^{59}Fe-citrate. Control (-FAC) and iron over-loaded (+FAC) mice were euthanized after 24 hours, and uptake of ^{59}Fe in major organs was quantified in a γ-counter (Supplementary Fig. 1).

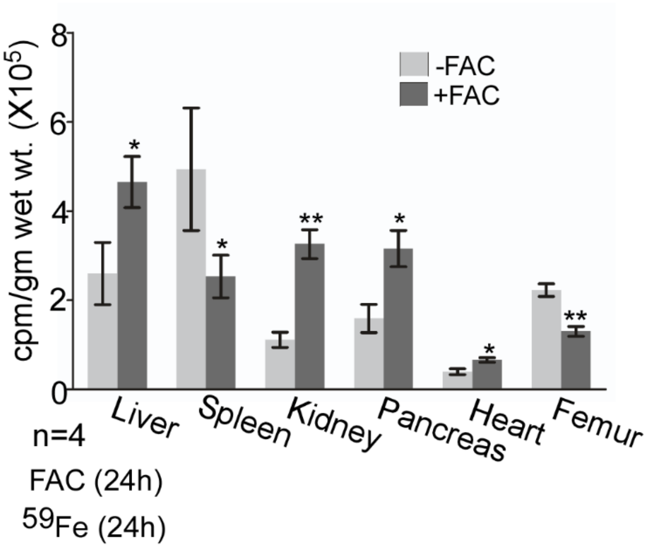

Supplementary Fig S1. The liver, kidney, pancreas, and heart take up 59Fe-NTBI, while the spleen and femur incorporate mainly Tf-Fe. Data represent the mean S.E. of the indicated n.*p<0.05**; p<0.01. Uptake of ^{59}Fe-NTBI by the liver [2], Fig 3 B, kidney [3] , Fig. 1 C, and pancreas (unpublished observations) of $PrP^{-/-}$ mice is significantly lower than $PrP^{+/+}$ controls.

Supplementary Fig S2: Estimation of C1 and C2 in mice pancreas

The C1 and C2 form was estimated in the mice pancreas by probing with 8H4. It was very evident that C2 form was more prominent in mice pancreas and the C1 was more in human brain.

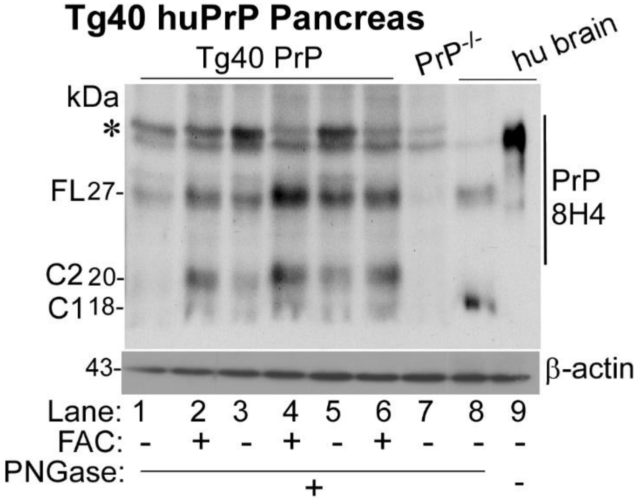

Supplementary Fig S2. Iron uptake is coupled with β-cleavage of PrP^{C}. Probing of deglycosylated pancreatic lysates from Tg40 mice with 8H4 shows β-cleavage of majority of PrP (lanes 1-6). Systemic iron overload increases FL and the β-cleaved form of PrP (lanes 2, 4 & 6 vs. 1, 3 & 5). Most of the PrP in human brain homogenate is C1 (lanes 8 & 9). Pancreatic lysates from $PrP^{-/-}$ mice show no reactivity for 8H4 as expected. * represents a non-specific band.

Supplementary Fig S3: Transfection efficiency in 1.1B4 cells

The transfection efficiency was analyzed by quantifying the expression of transfected proteins by manual counting. The expression of transfected proteins was similar in all treatments.

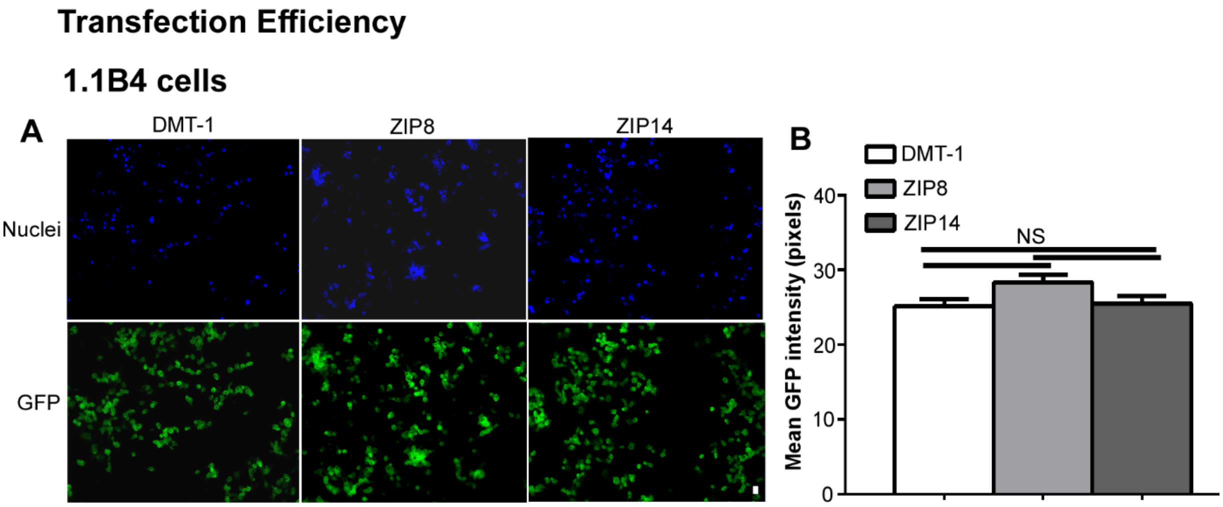

Supplementary Fig S3. The transfection efficiency was confirmed for analyzing the data in Figure 2G in the manuscript. **(A)** Fluorescence images acquired following transfection with DMT-1, ZIP-8 and ZIP-14

tagged GFP showed uniform transfection efficiency in all treatments which was further quantified using manual counting. Scale: 25µm. **(B)** Graphical representation of the mean GFP intensity was calculated using ImageJ. Five fields from each treatment was acquired (same number of cells analyzed), merged and presented. The mean GFP intensity was acquired for these images and no significant differences was observed between the treatments. Data represent the mean S.E. of the indicated n.*$p<0.05$ and NS represents no significant difference.

Supplementary Fig S4: PrP mediates iron uptake by pancreatic β-cells

Probing for ferritin revealed significantly less ferritin in $PrP^{-/-}$ relative to PrP controls. Overloading with iron caused significant upregulation of ferritin in Tg40 PrP, but not in $PrP^{-/-}$ samples. Expression of TfR was reduced in iron overloaded Tg40 PrP mice, but showed minimal change in similarly treated $PrP^{-/-}$ mice.

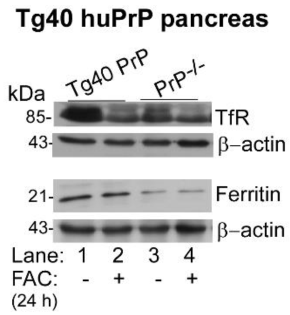

Supplementary Fig S4. PrP mediates iron uptake by pancreatic β-cells. Western blotting shows upregulation of PrP in iron-overloaded Tg40 PrP, no signal in $PrP^{-/-}$ samples. Ferritin is significantly higher in iron-overloaded relative to untreated Tg40 PrP samples and matched $PrP^{-/-}$ samples. There is no change in ferritin iron overloaded $PrP^{-/-}$ samples relative to untreated controls. Probing for TfR shows significant reduction in iron-overloaded relative to untreated Tg40 PrP samples and matched $PrP^{-/-}$ samples. There is minimal change in TfR expression in iron-overloaded $PrP^{-/-}$ samples relative to untreated controls.

Supplementary Fig S5: PrP-mediated increase in IC iron downregulates glucose transporters in the pancreas, brain, neuroretina, and the liver.

Probing of pancreatic and liver lysates for GLUT2, brain for GLUT-3 and retinal lysates for GLUT-1 revealed significantly higher expression in C6 $PrP^{-/-}$ relative to C6 $PrP^{+/+}$ samples. Overloading with iron downregulated GLUTs in C6 $PrP^{+/+}$, but had no influence on C6 $PrP^{-/-}$ mice

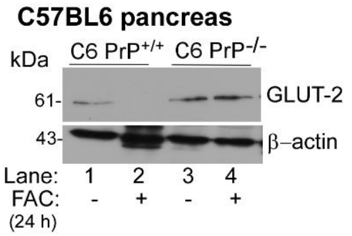

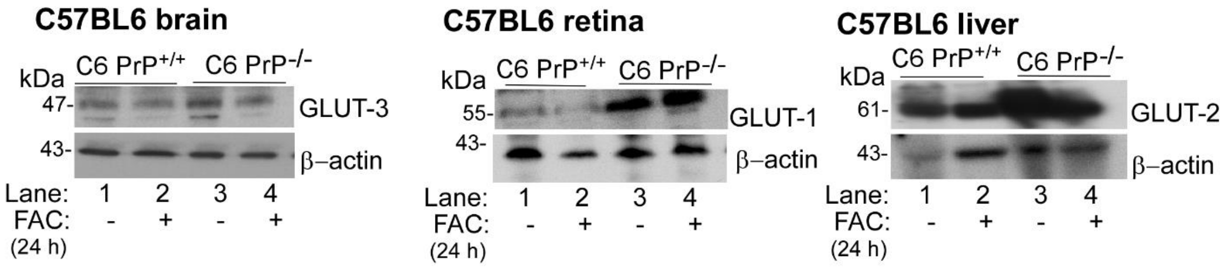

Supplementary Fig S5. PrP-mediated increase in IC iron downregulates glucose transporters in the pancreas, brain, neuroretina, and the liver. Probing of Western blots of lysates from C6 $PrP^{+/+}$ and C6 $PrP^{-/-}$ mice for GLUT-2, GLUT-3, GLUT-1 in pancreas/liver, brain and retina respectively shows a significant increase in C6 $PrP^{-/-}$ relative to C6 $PrP^{+/+}$ samples.

Supplementary Fig S6: PrP-mediated increase in IC iron downregulates insulin in the pancreas

Probing of pancreatic for insulin revealed significantly higher expression in C6 $PrP^{-/-}$ relative to C6 $PrP^{+/+}$ samples. Overloading with iron downregulated insulin in C6 $PrP^{+/+}$, but had no influence on C6 $PrP^{-/-}$ mice

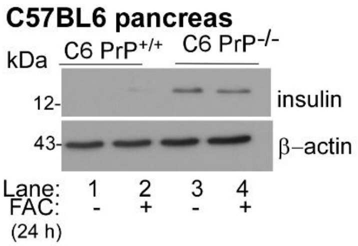

Supplementary Fig S6. PrP-mediated increase in IC iron downregulates insulin in the pancreas. Probing of Western blots of pancreatic lysates from C6 $PrP^{+/+}$ and C6 $PrP^{-/-}$ mice for insulin shows a significant increase in C6 $PrP^{-/-}$ relative to C6 $PrP^{+/+}$ samples.

Raw Data:

Below, we show complete gels of cropped protein bands. Relevant bands are marked with dotted lines. Some samples were not relevant to this study and were excluded.

Figure 1.

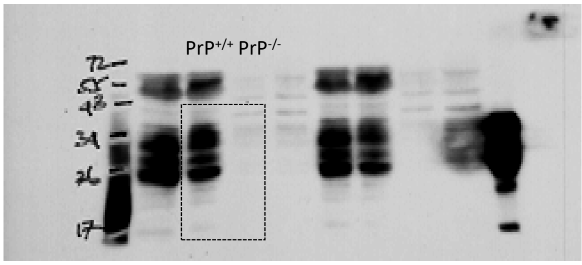

Figure 1B (order of probing: 1-PrP 8H4)

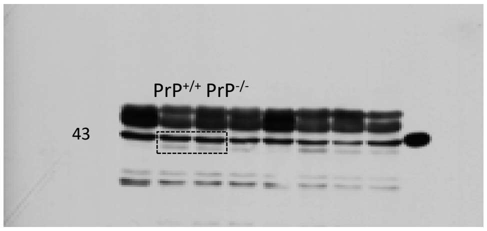

Figure 1B (order of probing: 1-PrP 8H4; 2- β-actin)

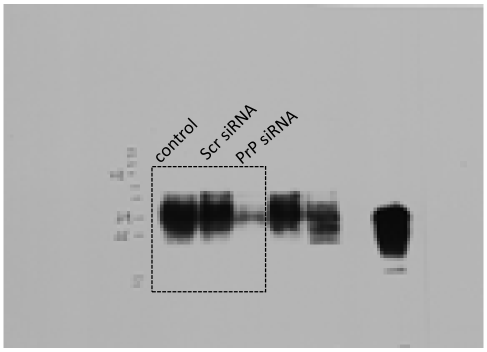

Figure 1C (order of probing: 1-PrP 3F4)

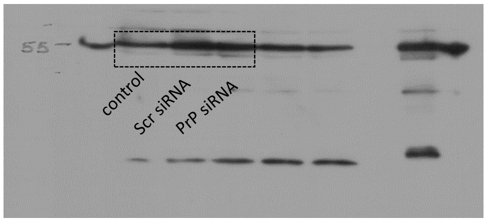

Figure 1C (order of probing: 1-PrP 3F4; 2- α-tubulin)

Figure 2

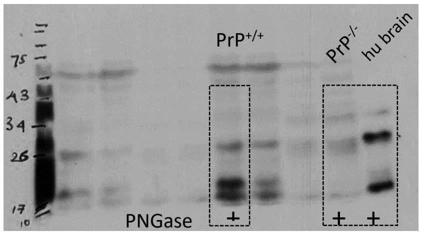

Figure 2A (order of probing: 1-PrP 8H4)

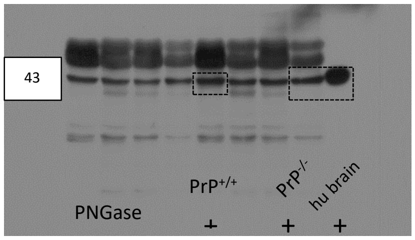

Figure 2A (order of probing: 1-PrP 8H4; 2- β-actin)

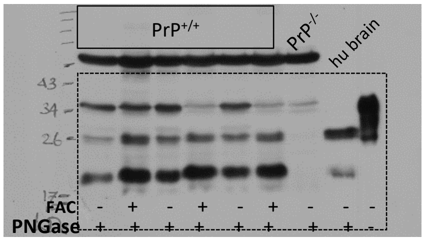

Figure 2B (order of probing: 1-PrP 3F4)

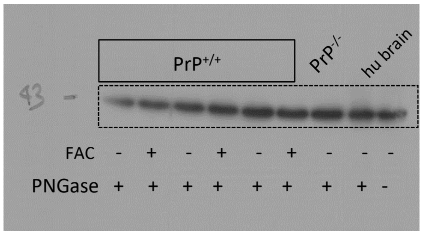

Figure 2B (order of probing: 1-PrP 3F4; 2- β-actin)

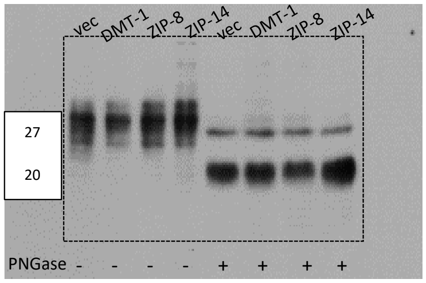

Figure 2F (order of probing: 1-PrP 3F4)

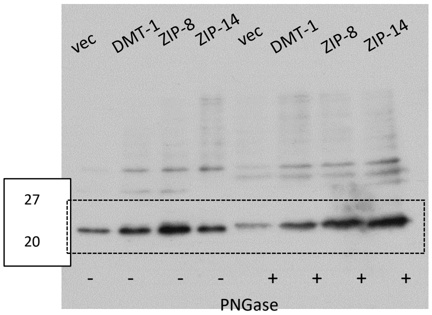

Figure 2F (order of probing: 1-PrP 3F4; 2- ferritin)

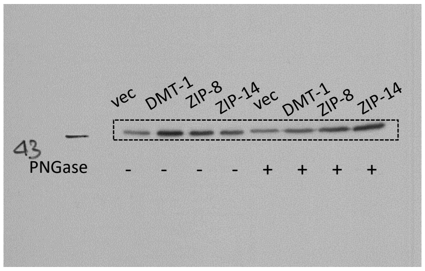

Figure 2F (order of probing: 1-PrP 3F4; 2-ferritin; 3- β-actin)

Figure 3

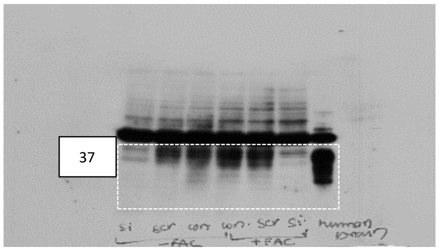

Figure 3A (order of probing: 1-PrP 3F4)

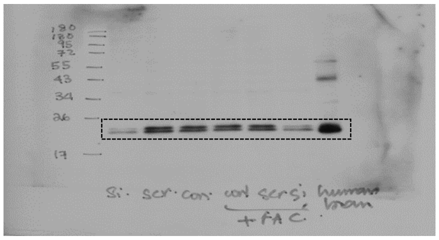
Figure 3A (order of probing: 1-PrP 3F4; 2- ferritin)

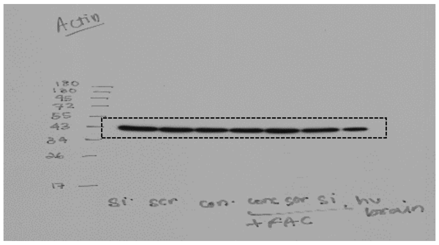
Figure 3A (order of probing: 1-PrP 3F4; 2-ferritin; 3- β-actin)

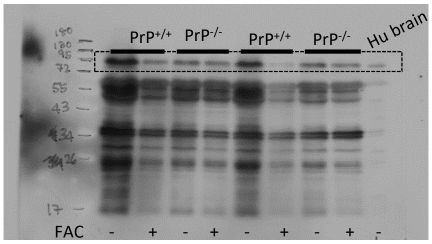

Figure 3C (order of probing: 1-TfR)

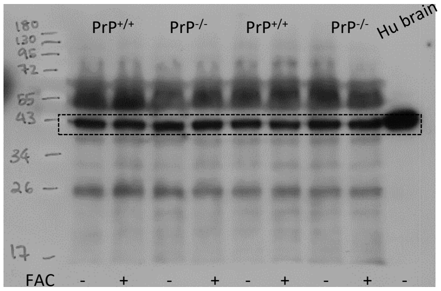

Figure 3C (order of probing: 1-TfR; 2- β-actin)

PrP$^{+/+}$

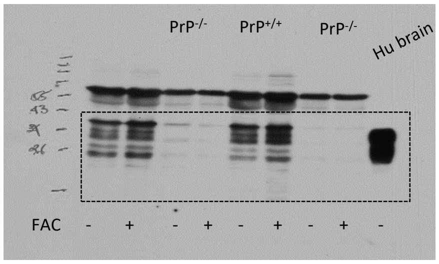

Figure 3C (order of probing: 1-PrP 8H4)

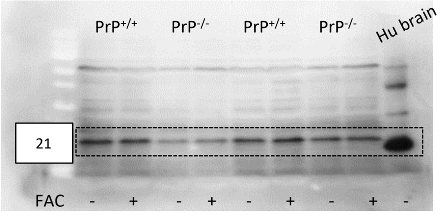

Figure 3C (order of probing: 1-PrP 8H4; 2-ferritin)

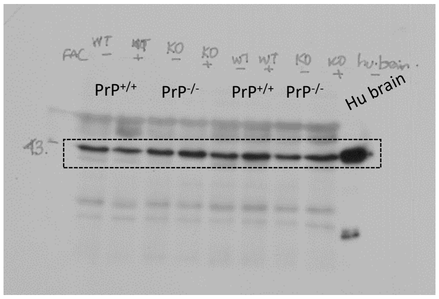

Figure 3C (order of probing: 1-PrP 8H4; 2-ferritin; 3-β-actin)

Figure 4

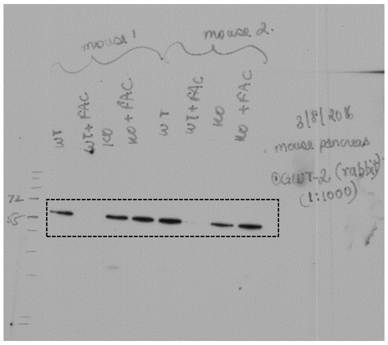

Figure 4A (order of probing: 1-GLUT-2)

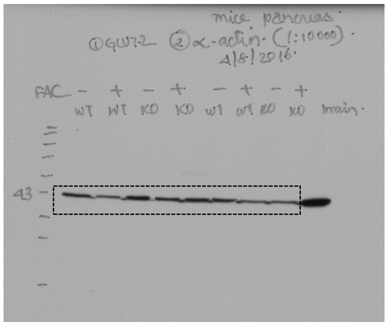

Figure 4A (order of probing: 1-GLUT-2; 2-β-actin)

Figure 5

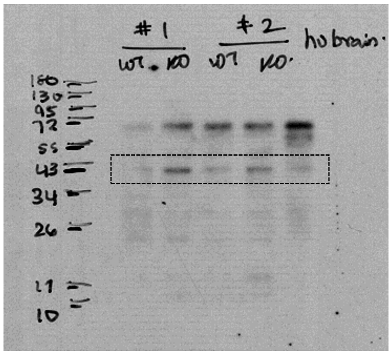

Figure 5A (order of probing: 1-GLUT-3)

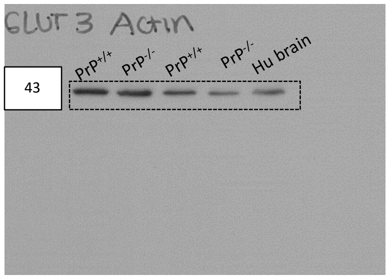

Figure 5A (order of probing: 1-GLUT-3; 2-β-actin)

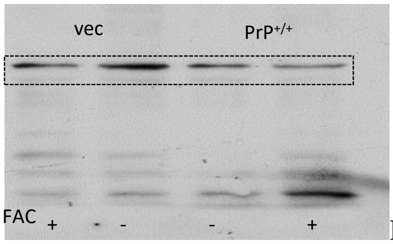

Figure 5D (order of probing: 1-GLUT-3)

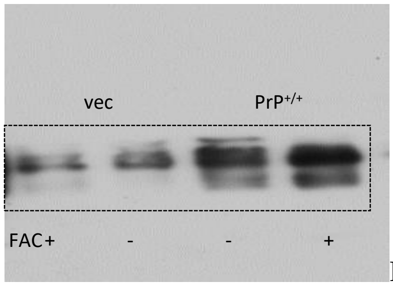

Figure 5D (order of probing: 1-GLUT-3; 2-PrP)

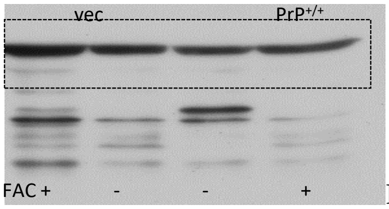

Figure 5D (order of probing: 1-GLUT-3; 2-PrP; 3-β-actin)

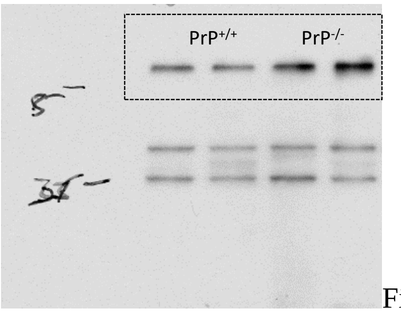

Figure 5F (order of probing: 1-GLUT-1)

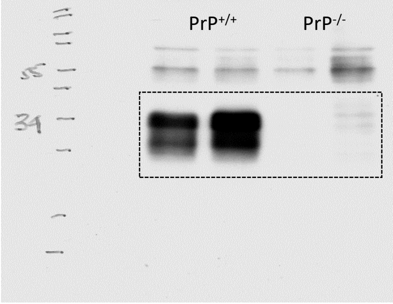

Figure 5F (order of probing: 1-GLUT-1; 2-PrP)

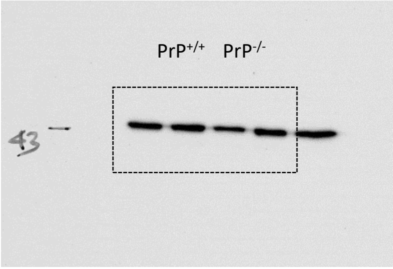

Figure 5F (order of probing: 1-GLUT-1; 2-PrP; 3-β-actin)

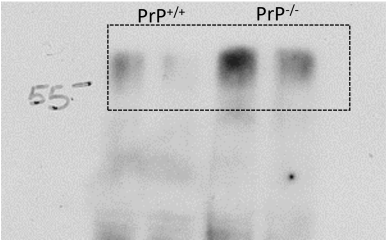

Figure 5H (order of probing: 1-GLUT-2)

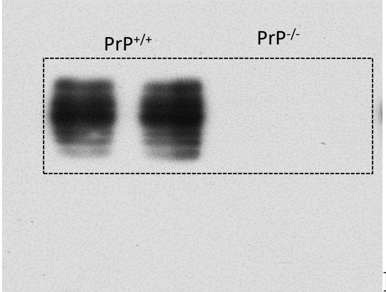

Figure 5H (order of probing: 1-GLUT-2; 2-PrP)

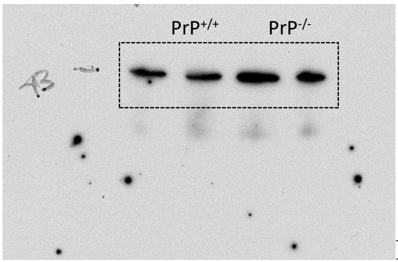

Figure 5H (order of probing: 1-GLUT-2; 2-PrP; 3-β-actin)

Figure 6

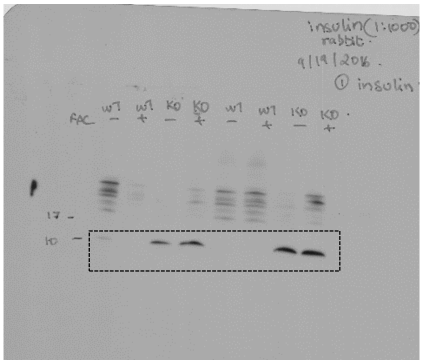

Figure 6A (order of probing: 1-insulin)

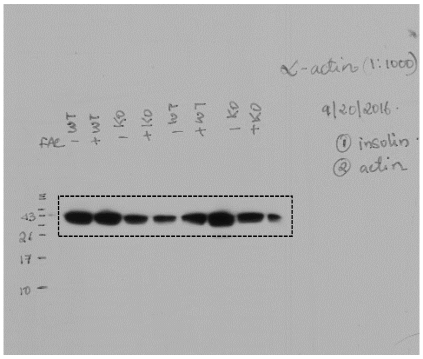

Figure 6A (order of probing: 1-insulin; 2- β-actin)

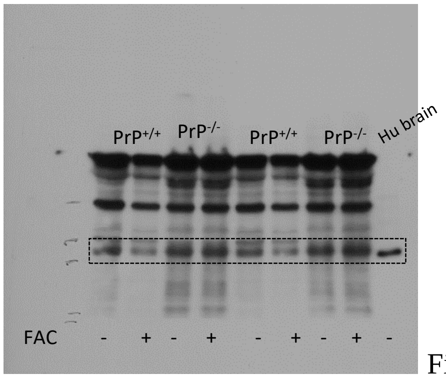

Figure 6A (order of probing: 1-insulin)

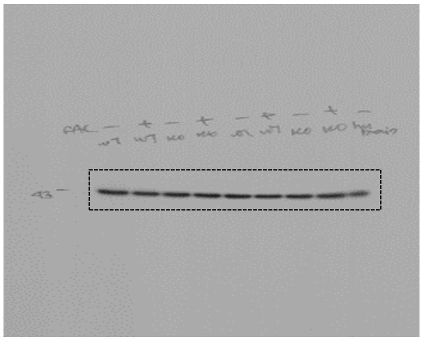

Figure 6A (order of probing: 1-insulin; 2- β-actin)

References

[1] C.M. Craven, J. Alexander, M. Eldridge, J.P. Kushner, S. Bernstein, J. Kaplan, Tissue distribution and clearance kinetics of non-transferrin-bound iron in the hypotransferrinemic mouse: a rodent model for hemochromatosis, Proceedings of the National Academy of Sciences of the United States of America 84(10) (1987) 3457-61.

[2] A.K. Tripathi, S. Haldar, J. Qian, A. Beserra, S. Suda, A. Singh, U. Hopfer, S.G. Chen, M.D. Garrick, J.R. Turner, Prion protein functions as a ferrireductase partner for ZIP14 and DMT1, Free Radical Biology and Medicine 84 (2015) 322-330.

[3] S. Haldar, A. Tripathi, J. Qian, A. Beserra, S. Suda, M. McElwee, J. Turner, U. Hopfer, N. Singh, Prion protein promotes kidney iron uptake via its ferrireductase activity, Journal of Biological Chemistry 290(9) (2015) 5512-5522.
